# Denoising Improves Cross‐Scanner and Cross‐Protocol Test–Retest Reproducibility of Diffusion Tensor and Kurtosis Imaging

**DOI:** 10.1002/hbm.70142

**Published:** 2025-03-07

**Authors:** Benjamin Ades‐Aron, Santiago Coelho, Gregory Lemberskiy, Jelle Veraart, Steven H. Baete, Timothy M. Shepherd, Dmitry S. Novikov, Els Fieremans

**Affiliations:** ^1^ Bernard and Irene Schwartz Center for Biomedical Imaging, Department of Radiology New York University School of Medicine New York New York USA; ^2^ Microstructure Imaging Inc. Brooklyn New York USA

**Keywords:** brain white matter, clinical translation, diffusion kurtosis imaging, diffusion tensor imaging, higher‐order diffusion, image denoising, MPPCA, test–retest reproducibility

## Abstract

The clinical translation of diffusion magnetic resonance imaging (dMRI)‐derived quantitative contrasts hinges on robust reproducibility, minimizing both same‐scanner and cross‐scanner variability. As multi‐site data sets, including multi‐shell dMRI, expand in scope, enhancing reproducibility across variable MRI systems and MRI protocols becomes crucial. This study evaluates the reproducibility of diffusion kurtosis imaging (DKI) metrics (beyond conventional diffusion tensor imaging (DTI)), at the voxel and region‐of‐interest (ROI) levels on magnitude and complex‐valued dMRI data, using denoising with and without harmonization. We compared same‐scanner, cross‐scanner, and cross‐protocol variability for a multi‐shell dMRI protocol (2‐mm isotropic resolution, *b* = 0, 1000, 2000 s/mm^2^) in 20 subjects. We first evaluated the effectiveness of Marchenko‐Pastur Principal Component Analysis (MPPCA) based denoising strategies for both magnitude and complex data to mitigate noise‐induced bias and variance, to improve dMRI parametric maps and reproducibility. Next, we examined the impact of denoising under different population analysis approaches, specifically comparing voxel‐wise versus region of interest (ROI)‐based methods. We also evaluated the role of denoising when harmonizing dMRI across scanners and protocols. The results indicate that DTI and DKI maps visually improve after MPPCA denoising, with noticeably fewer outliers in kurtosis maps. Denoising, either using magnitude or complex dMRI, enhances voxel‐wise reproducibility, with test–retest variability of kurtosis indices reduced from 15%–20% without denoising to 5%–10% after denoising. Complex dMRI denoising reduces the noise floor by up to 60%. Denoising not only reduced variability across scans and protocols, but also increased statistical power for low SNR voxel‐wise comparisons when comparing cross sectional groups. In conclusion, MPPCA denoising, either over magnitude or complex dMRI data, enhances the reproducibility and precision of higher‐order diffusion metrics across same‐scanner, cross‐scanner, and cross‐protocol assessments. The enhancement in data quality and precision facilitates the broader application and acceptance of these advanced imaging techniques in both clinical practice and large‐scale neuroimaging studies.

## Graphical abstract

MPPCA denoising enhances the reproducibility and precision of higher‐order diffusion metrics in dMRI, by reducing variability and noise across same‐scanner, cross‐scanner, and cross‐protocol assessments. This improvement supports broader clinical application and acceptance of advanced imaging techniques.

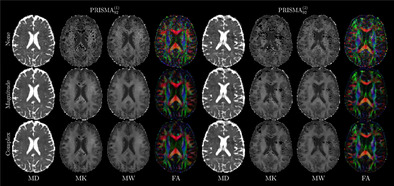

**Abbreviations**
ABCD: adolescent brain cognitive development
ADNI: Alzheimer's disease neuroimaging initiative
CCC: concordance correlation coefficient
CV: coefficient of variation
DKI: diffusion kurtosis imaging
dMRI: diffusion magnetic resonance imaging
DTI: diffusion tensor imaging
HCP: Human Connectome Project
ICC: intraclass correlation coefficient
MPPCA: Marchenko‐Pastur principal component analysis
ROI: region of interest

## 1 Introduction

Diffusion magnetic resonance imaging (dMRI) is a noninvasive method enabling clinicians and researchers to characterize tissue microstructure beyond the nominal resolution of MRI. It measures the restricted diffusion of water within tissues (Jones and Diffusion 2010), providing insights into neurological structure and function (Alexander et al. 2019; Jelescu and Budde 2017; Novikov, Kiselev, and Jespersen 2018). Recent large scale data initiatives like the Alzheimer's Disease Neuroimaging Initiative (ADNI) (Jack Jr et al. 2008), Adolescent Brain Cognitive Development (ABCD) (Jack Jr et al. 2008), the Human Connectome Project (HCP) (Van Essen et al. 2013), and UK Biobank (Sudlow et al. 2015), aim to utilize multi‐shell dMRI to achieve a more comprehensive characterization of brain microstructure than possible with conventional diffusion tensor imaging (DTI). Higher‐order diffusion signal representations and biophysical models (Alexander et al. 2019; Jelescu and Budde 2017; Novikov et al. 2019; Novikov, Kiselev, and Jespersen 2018) have been shown to characterize age related tissue changes in healthy populations (Beck et al. 2021; Cox et al. 2016; Novikov et al. 2019) and pathology stemming from disease (Dong et al. 2020; Falangola et al. 2013; de Kouchkovsky et al. 2016; Pogosbekian et al. 2021; Stauffer et al. 2021).

Despite its potential, the variability of dMRI‐derived parameters, across different scanners and even on the same scanner, challenges its translation into clinical practice (Vollmar et al. 2010). This variability stems from factors like changing nuclear magnetic resonance (NMR) contrast (e.g., due to operating at different echo time or field strength), imaging artifacts like bias due to motion or Gibbs ringing, and MRI noise which introduces random fluctuations and bias from the non‐central‐χ MRI noise floor (Aja‐Fernández, Tristán‐Vega, and Alberola‐López 2009; Gudbjartsson and Patz 1995). These artifacts, coupled with poor noise propagation through the high‐order representations (such as diffusion kurtosis imaging [DKI] (Jensen et al. 2005)), lead to large outliers in quantitative maps (Sprenger et al. 2016), further reducing the reproducibility of such parameters. These variabilities limit dMRI to a qualitative rather than a quantitative modality, particularly as MRI technology itself rapidly advances, prompting frequent updates to study protocols (such as parallel imaging (Griswold et al. 2002; Sodickson and Manning 1997; Pruessmann et al. 1999) and simultaneous multislice [SMS] imaging (Setsompop et al. 2012)) that affect NMR contrast and noise floors. Low signal‐to‐noise‐ratio (SNR) reduces the precision of dMRI and amplifies scanner‐ and protocol‐dependent biases (Jones and Basser 2004), necessitating effective noise suppression strategies (Does et al. 2019; Manzano Patron et al. 2024) to enhance data reliability, harmonization, and enable more accurate comparisons of diffusion parameters between healthy and pathological tissues.

Denoising is a critical component of diffusion MRI (dMRI) preprocessing, aimed at minimizing noise while preserving the integrity of diffusion measurements. Principal component analysis (PCA)‐based techniques, such as Marchenko‐Pastur principal component analysis (MPPCA) (Veraart, Fieremans, and Novikov 2016; Veraart et al. 2016) and local PCA (LPCA) (Manjón et al. 2013), are widely employed to separate signal from noise by leveraging redundancy in diffusion data across spatial and *q*‐space dimensions. Filter‐based methods like BM4D (Maggioni et al. 2012) and non‐local means (NLM) (Manjon et al. 2010) utilize non‐local patch matching to enhance noise reduction while maintaining high fidelity to the underlying anatomical structures. More recently, machine learning approaches like Patch2Self (Fadnavis, Batson, and Garyfallidis 2020) and convolutional neural network (CNN) methods (Tian et al. 2020, 2022) have been applied to dMRI, leveraging neural networks to perform self‐supervised denoising, allowing for more effective noise suppression without requiring external training data.

In this study, we evaluate two noise reduction techniques based on Random Matrix Theory—MPPCA for both magnitude and complex image data—to reduce within‐scanner, cross‐scanner, and cross‐protocol variability. We compare *repeatability* (pure test–retest comparisons without changing protocol or hardware) and *reproducibility* (comparisons of same‐subject data while varying protocol/hardware conditions) of three kinds of processing: (i) without denoising; (ii) using state‐of‐the‐art MPPCA denoising (Veraart, Fieremans, and Novikov 2016, Veraart et al. 2016) for magnitude images; and (iii) adopting MPPCA to denoise complex images (Henriques et al. 2023; Lemberskiy et al. 2019), to maximize the precision of conventional DTI and higher‐order diffusion metrics. MPPCA identifies an optimal threshold for noise‐only singular values using the Marchenko‐Pastur (MP) distribution. This technique and its variations have become a widely used component of dMRI pre‐processing pipelines (Cordero‐Grande et al. 2019; Moeller et al. 2021; Olesen et al. 2023). Complex MPPCA operates on complex‐valued data (after coil combination), and is enabled through phase estimation and unwinding prior to applying the MPPCA algorithm (Cordero‐Grande et al. 2019; Grussu et al. 2020).

We scanned 20 human subjects using in total five dMRI protocols on two different MRI scanners (same vendor but with different gradient hardware). This unique dataset includes for each subject scan‐rescan measurements on both scanners using a dMRI protocol optimized for SNR by minimizing echo time for the specific hardware, as well as cross‐scanner measurements using the same dMRI protocol on both scanners, with a dMRI protocol optimized for the scanner with the weaker gradients. By comparing denoising strategies across these measurements with varying SNR, we sought to determine how MPPCA denoising can mitigate SNR‐related biases and improve the consistency of diffusion metric quantification, with the ultimate goal of enhancing reproducibility across varying hardware and imaging protocols.

## 2 Methods

### 2.1 Clinical MRI Protocol

This HIPAA‐compliant prospective study was approved by the local institutional review board. After providing informed consent, 20 healthy volunteers (10 male/10 female, age = 32.2 ± 9.7 years) underwent brain dMRI on Siemens Magnetom Prisma and Skyra 3T systems. All protocols included a diffusion weighted monopolar spin echo EPI sequence with 2‐mm isotropic resolution, using a 20‐channel head coil for reception and *b*‐values commonly used in multi‐shell acquisitions (Sudlow et al. 2015; Van Essen et al. 2013) including *b* = 0, 1000, and 2000 s/mm shells, as detailed in Table 1. Acceleration was performed using 6/8 partial Fourier, in‐plane parallel imaging (GRAPPA factor 2), and simultaneous multislice (SMS) acceleration with multiband factor 2. Raw data from all acquisitions were saved in the Siemens TWIX data format and fed into a standard reconstruction pipeline implemented in MATLAB for the purpose of saving phase maps. The pipeline (in order of processing steps) included coil noise decorrelation (Brey and Narayana 1988), Nyquist ghost correction (Ahn and Cho 1987), slice‐wise GeneRalized Autocalibrating partially parallel acquisitions (GRAPPA) (Griswold et al. 2002; Larkman et al. 2001), Controlled Aliasing In Parallel Imaging Results IN Higher Acceleration (CAIPIRINHA) shift (Breuer et al. 2005), in‐plane GRAPPA, trapezoidal regridding (Bernstein, King, and Zhou 2004), adaptive combine (Walsh, Gmitro, and Marcellin 2000), and Projection onto Complex Sets (POCS) (Amartur and Haacke 1991). During this reconstruction, both magnitude and phase images were saved for use during subsequent denoising and artifact correction steps.

**TABLE 1:** The three dMRI protocols used during this study. Each of the 20 subjects underwent these three protocols to acquire in total five datasets, including a test–retest dataset on the Prisma, test–retest on the Skyra, and a single dataset acquired on the Prisma with protocol matched to the Skyra.

Scanner	Number of datasets	Dataset labels	TR (ms)	TE (ms)	*b* = 0 s/mm	*b* = 1000 s/mm^2^	*b* = 2000 s/mm^2^	Acq. time (min:s)	Gradient (mT/m)	Software version	Coils
Prisma	2	$P_{92}^{\left(1,2\right)}$	5300	92	5	20	40	7:28	80	VEC11	20 channel head
Prisma	1	$P_{127}^{\left(1\right)}$	6700	127	5	15	30	7:28	80	VEC11	20 channel head
Skyra	2	$S_{127}^{\left(1,2\right)}$	6700	127	5	15	30	7:27	40	VEC11	20 channel head

Five test–retest dMRI datasets were acquired for each subject, as listed in Table 1. Two test–retest datasets were acquired on the Prisma system using the dMRI protocol with the shortest possible TE = 92 ms. A third dataset was acquired on the Prisma using a modified protocol (with TE = 127 ms) to study cross‐protocol reproducibility. Next, two datasets were acquired on the Skyra system using the dMRI protocol with the shortest possible TE = 127 ms (due to smaller maximal gradient). Scan time for all dMRI protocols (subset used here) was approximately the same at 7.5 min, with respectively 60 and 50 directions on the Prisma and Skyra systems.

Between test–retest acquisitions, subjects were removed and then placed back into the scanner. Each test–retest dataset included a reverse phase encoding *b* = 0 image to correct for EPI‐induced distortions. A 1‐mm isotropic T1‐weighted Magnetization Prepared Rapid Gradient Echo (MP‐RAGE) sequence (TR/TE/TI = 2200/3.17/900 ms) was acquired on the Prisma system for co‐registration of all five datasets per subject.

These five datasets were used to perform three comparisons.

1. Effect of denoising on *within‐scanner variability* (*repeatability*) by comparing Prisma versus rescan Prisma^(2)^ with TR/TE 5300/92 ms (P^(1)^_92_ vs. P^(2)^_92_) and comparing Skyra^(1)^ versus rescan Skyra^(2)^ with TR/TE = 6700/127 ms, referred to as $S_{127}^{\left(1\right)}$ versus $S_{127}^{\left(2\right)}$.
2. Effect of denoising on *cross‐protocol variability* (*reproducibility*) by comparing data from the same scanner with unmatched TE and SNR: Prisma^(1)^ TR/TE = 5300/92 ms versus Prisma^(1)^ TR/TE = 6700/127 ms, referred to as $P_{92}^{\left(1\right)}$ vs. $P_{127}^{\left(1\right)}$.
3. Effect of denoising on *inter‐scanner variability* (*reproducibility*) by comparing data from different scanners, but matched TE: Skyra^(1)^ TR/TE = 6700/127 ms versus Prisma TR/TE = 6700/127 ms, referred to as $S_{127}^{\left(1\right)}$ versus $P_{127}^{\left(1\right)}$.

### 2.2 MPPCA‐Based Denoising Methods

Denoising was performed using an augmentation of the MPPCA algorithm (Veraart, Fieremans, and Novikov 2016; Veraart et al. 2016). MPPCA exploits data redundancy in the singular value decomposition (SVD)/principal component analysis (PCA) domain using properties of the eigenspectrum of random covariance matrices, which yields an objective number p of signal‐carrying components to be kept and all other components removed as purely noise‐carrying.

A data matrix $X=\left\{X_{\mathrm{mn}}\right\}\equiv \left\{S_{m}\left(r_{n}\right)\right\}$ of size *M* × *N* is formed by *M* measurements from *N* voxels in a patch. Let $M^{\prime }$ = min(*M*, *N*) and $N^{\prime }$ = max(*M*, *N*). Low‐rank denoising corresponds to keeping p largest components in the SVD of $X=\sum_{i=1}^{M^{\prime }}s_{i}u_{i}⊗v_{i}$, where $u_{i}$ and $v_{i}$ are the left and right singular vectors. Equivalently, PCA corresponds to keeping p top eigenvalues $x_{i}=s_{i}^{2}$ explaining most of the variance of the (sample) covariance matrix $XX^{⊤}$. MPPCA yields the number p of the components of *X* to keep, by identifying the MP distribution formed by the remaining $M^{\prime }-p$ components that correspond to pure noise in the limit *M*, *N* ≫ 1 and p ≪ *M*, *N* (low‐rank condition). MPPCA self‐consistently finds *p*, and noise variance σ^2^ as the sum over the $M^{\prime }-p$ components attributed to the MP distribution.

In recent years there having been several proposed additions to the original MPPCA algorithm, including symmetric thresholding of singular values (Olesen et al. 2023), eigenvalue shrinkage (Gavish and Donoho 2017), advanced patching methods including non‐local spatial matching (Buades, Coll, and Morel 2005; Maggioni et al. 2012; Manjón, Coupé, and Buades 2015; Zhao et al. 2022), angular matching (St‐Jean, Coupé, and Descoteaux 2016), and structural adaptation (Bao et al. 2013). Here, MPPCA is augmented in three stages: (i) Adaptive patching to select the signals forming a data matrix *X* around a given voxel; (ii) Symmetric SVD threshold selection (Olesen et al. 2023); (iii) Singular value shrinkage (Gavish and Donoho 2017). Here we carefully chose augmentations to MPPCA to balance spatial redundancy, signal similarity, and noise reduction efficiency. For additional information about available tunable parameters in MPPCA, users can view the software documentation available online at https://nyu‐diffusionmri.github.io/DESIGNER‐v2/docs/designer/background. We here describe each stage:

- *Adaptive patching—*In contrast to the original MPPCA approach where patches are squares or cubes around a given voxel (e.g., 5 × 5 × 5 voxels), we enhanced the spatial redundancy, in addition to the redundancy in the measurements (here in the diffusion *q*‐space), by pre‐selecting voxels that have similar ground truth. Our goal is to minimize the number p of components, and to maximize their contributions $s_{i}$, such that they describe most of the signal—this is the assumption of the underlying noise‐free signal to be of low‐rank. The ultimate best choice of the patch would be having all *N* voxels with the same ground truth; in this case, *p* ≡ 1 (the rank of noise‐free *XX* ^⊤^ is 1), irrespective of the complexity of their signal *S*_*m*_. The desire to have a few large $s_{i},\;i=1,\ldots ,p$ (as opposed to many smaller ones) comes from the fact that components whose noise‐free singular values $s_{i}^{\left(0\right)}<s_{*}=\sigma {\left(\mathrm{MN}\right)}^{1/4}$ are below the threshold $s_{*}$ become indistinguishable from noise. They fall below what's referred to as the *phase transition* (Johnstone 2007). By selecting a subset of voxels that have similar underlying tissue priors, we intend to overcome the information loss associated with many components potentially falling under the phase transition threshold s_*_, if all voxels in a patch were to have different signals.

Hence, for a voxel at $r_{0}$, we would like to include the signals $S_{m}\left(r_{n}\right)$ such that both $S_{m}$ are close to each other for different $r_{n}$, and the voxels $r_{n}$ are not too far from $r_{0}$. Formally, we introduce the “distance” between signals

(1) $$w_{\alpha ,\beta }\left[S\left(r\right),S\left(r^{\prime }\right)\right]={\left|r-r^{\prime }\right|}^{\alpha }\cdot {\left|S\left(r\right)-S\left(r^{\prime }\right)\right|}^{\beta }$$

**FIGURE 1:**
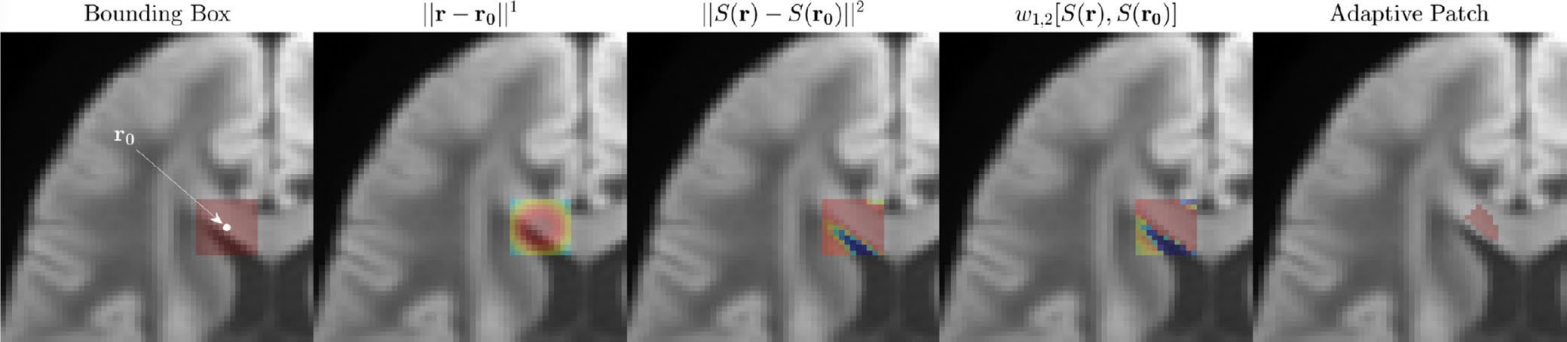
Example of how distances $w_{\alpha ,\beta }\left[S\left(r_{n}\right),S\left(r_{0}\right)\right]$ Equation (1), are generated for adaptive patching. These distances are used to threshold a cubic patch into the shape of local anatomy. We first choose a sufficiently large bounding box (in this example an 11 × 11 × 11 bounding box was chosen for demonstration purposes, in the study a 7 × 7 × 7 bounding box was used). Next, the Euclidean distances between voxels (2nd panel) and signals (3rd panel) are calculated. The weights (1) are computed based on the product of the two distances, tuned by exponents *α* and *β*. The patch selection corresponding to *N* = 100 is shown in the last panel.

Here $|r-r^{\prime }|$ is the Euclidean distance between voxels in three dimensions, and $|S\left(r\right)-S\left(r^{\prime }\right)|=\sqrt{\sum_{m}{\left|S_{m}\left(r\right)-S_{m}\left(r^{\prime }\right)\right|}^{2}}$ is the Euclidean distance between signals (the norm over the measurement index *m*). The balance of preferring the distance between voxels and between signals is tuned by the choice of exponents *α* and *β*. Here we choose *α* = 1 and *β* = 2 based on an empirical observation of improved denoising performance when *β* > *α* (preferring similarity of signals to the distance from $r_{0}$). When *α* ≫ *β* this method converges to the original MPPCA patching implementation (local signal‐independent patch around $r_{0}$ ). Based on the above distance function, we choose a patch around voxel at $r_{0}$ as *N* voxels (including $r_{0}$ ), for which $w_{1,2}\left[S\left(r_{n}\right),S\left(r_{0}\right)\right]$ is the smallest; here *N* was fixed to 100, Figure 1, selected from each larger 7×7×7 cubic patch.

ii *Symmetric thresholding—*For the pure‐noise case of p = 0, all components of *XX* ^⊤^ form the MP distribution, which has two independent properties: $\sigma^{2}=\sum x_{i}/\left(\mathrm{MN}\right)$ and $\sigma^{2}=\left(x_{1}-x_{M^{\prime }}\right)/4\sqrt{\mathrm{MN}}$. For a general case (noisy signal), MPPCA uses these two properties to define two functions $\sigma_{1,2}\left(p\right)$ accounting for the noise variance from the bottom $M^{\prime }-p$ components, with p being the solution for $\sigma_{1}\left(p\right)=\sigma_{2}\left(p\right)$. Here, we use the following definitions (Cordero‐Grande et al. 2019; Olesen et al. 2023):

(2.1) $$\begin{array}{c} \sigma_{1}^{2}\left(p\right)=\frac{1}{\left(N^{\prime }-p\right)\left(M^{\prime }-p\right)}\sum_{i=p+1}^{M^{\prime }}x_{i} \end{array}$$

(2.2) $$\begin{array}{c} \sigma_{2}^{2}\left(p\right)=\frac{x_{p+1}-x_{M^{\prime }}}{4\sqrt{\left(N^{\prime }-p\right)\left(M^{\prime }-p\right)}} \end{array}$$

These are symmetric in $M^{\prime }$ and $N^{\prime }$, whereas the original MPPCA formulation had $N^{\prime }$ instead of $N^{\prime }-p$ in Equation (2). This symmetric augmentation of $\sigma_{1,2}\left(p\right)$ empirically provides a more robust estimation of MP threshold for not very large *N* and *M*, and works well for *M*≈*N*. It is also practically important that the patch size is allowed to vary, with possibilities of both *M* > *N* and *M* < *N*. In this study *N* was fixed at 100, and *M* was the total number of diffusion measurements for all subjects and scan–rescan datasets. *M* = 65 for $P_{92}^{\left(1,2\right)}$, *M* = 50 for $S_{127}^{\left(1,2\right)}$, and *M* = 50 for $P_{127}^{\left(1\right)}$.

iii *Singular value shrinkage—*To overcome the eigenvalue repulsion due to noisy components, we reduce the sample singular values $s_{i}$ when recombining selected principal components into a low‐rank matrix $\hat{X_{\eta }}=\sqrt{M^{\prime }}\sigma \sum_{i=1}^{p}\eta \left(s_{i}/\left(\sqrt{M^{\prime }}\sigma \right)\right)\;\;u_{i}⊗v_{i}$. According to Gavish and Donoho (2017), the optimal shrinker function based on minimizing MSE of the Frobenius norm can be expressed as

(3)

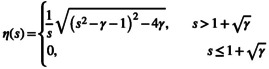

where $\gamma =M^{\prime }/N^{\prime }\leq 1$.

### 2.3 Denoising Experiments

dMRI raw data was preprocessed in three different ways for comparison:

- Magnitude (non‐denoised) dMRI data.
- MPPCA: Magnitude dMRI data were MPPCA‐denoised (including the adaptive patch, symmetric thresholding, eigenvalue shrinkage).
- MP‐Complex: dMRI phases were first denoised using MPPCA (symmetric thresholding) using a 15 × 15 2D box‐shaped kernel within each slice. The denoised and spatially smoothly varying phase $\phi_{\mathrm{dn}}\left(r\right)$ is then unwound according to: Sreal=ReScomplexe−iϕdn. Finally, the noisy phase‐unwound signal $S_{\text{real}}$ is denoised using an adaptive 3D moving patch, symmetric thresholding, and eigenvalue shrinkage. Phase unwinding helps remove spurious components arising due to shot‐to‐shot phase variations in dMRI (Grussu et al. 2020; Lemberskiy et al. 2019). In addition to improved denoising performance (due to the two‐pass approach), MP‐Complex also reduces the Rician noise floor of the data, reducing bias and increasing the precision of downstream parameter estimation.

### 2.4 Processing Pipeline and Diffusion Parameter Estimation

All dMRI images were processed using the DESIGNER pipeline (Ades‐Aron et al. 2018; Chen et al. 2024), using Partial Fourier induced Gibbs artifact correction (Kellner et al. 2016; Lee, Novikov, and Fieremans 2021), EPI distortion correction (Smith et al. 2004), and eddy‐current and motion correction (including slice‐wise outlier replacement) (Andersson et al. 2016; Andersson and Sotiropoulos 2016). The signal's normalized rotational invariants of zeroth (*S*_0_, also known as spherical mean) order were linearly estimated for each diffusion shell (Novikov, Veraart, et al. 2018) to provide a convenient basis for population‐wise registration.

Diffusion and kurtosis tensors were estimated through unconstrained weighted linear least squares (Veraart et al. 2013) from which we derived mean diffusivity (MD), axial diffusivity (AD), radial diffusivity (RD), fractional anisotropy (FA), mean kurtosis (MK), axial kurtosis (AK), and radial kurtosis (RK). DTI parameters (e.g., FA and MD) were derived specifically from a DTI fit, utilizing only the *b* = 0 and *b* = 1000 s/mm (Jelescu and Budde 2017) diffusion shells. These parameters are not derived from the DKI fit, where additional higher *b* values are included. This distinction is important, as the reproducibility of DTI parameters derived from a DTI‐specific fit may differ from those derived from a DKI fit. For completeness we also show ROI means for DTI parameters derived from the DKI fit in Figure S4.

The minimal protocol used in this study uses kurtosis estimation from the cumulant expansion up to $O\left(b^{2}\right)$:

(4) $$\begin{array}{c} \ln\;\frac{S\left(b,\hat{n}\right)}{{\left.S\right|}_{b=0}}=-\mathrm{bD}\left(\hat{n}\right)+\frac{1}{6}b^{2}{\bar{D}}^{2}W\left(\hat{n}\right)+\ldots \end{array}$$

where $D\left(\hat{n}\right)=D_{\mathrm{ij}}\;n_{i}n_{j}$, $W\left(\hat{n}\right)=W_{\text{ijkl}}\;n_{i}n_{j}n_{k}n_{l}$, and

(5) $$\begin{array}{c} \mathrm{MD}\equiv \bar{D}=\int_{\left|\hat{n}\right|=1}\frac{d\hat{n}}{4\pi }\;D\left(\hat{n}\right)=\frac{1}{3}D_{\mathrm{ii}} \end{array}$$

(the Einstein's convention of summation over pairs of repeated indices is assumed throughout).

The definition of mean kurtosis is ambiguous in the dMRI literature. The original paper of Jensen et al. (2005) suggested that MK is an angular average of the directional kurtosis $K\left(\hat{n}\right)={\bar{D}}^{2}W\left(\hat{n}\right)/D^{2}\left(\hat{n}\right):$

(6) $$\begin{array}{c} \mathrm{MK}={\bar{D}}^{2}\int_{\left|\hat{n}\right|=1}\frac{d\hat{n}}{4\pi }\;\frac{W\left(\hat{n}\right)}{D^{2}\left(\hat{n}\right)}\; \end{array}$$

This definition emphasizes the directional character of the kurtosis as opposed to that of the cumulant, However, this definition has two notable drawbacks. First, fundamentally, the result cannot be compactly represented as a trace of a certain tensor (e.g., analogously to Equation 5)—due to a nontrivial directional dependence of the denominator. Second, practically, this definition leads to a relatively low precision and is strongly affected by outliers, which come from directions where diffusivity $D\left(\hat{n}\right)\ll \bar{D}$ is small.

In this work, we adopt the definition that involves angular average of the cumulant (Hansen et al. 2013):

(7) $$\begin{array}{c} \mathrm{MW}\equiv \bar{W}=\int_{\left|\hat{n}\right|=1}\frac{d\hat{n}}{4\pi }\;W\left(\hat{n}\right)=\frac{1}{5}\;W_{\text{iikk}}\; \end{array}$$

The last equation follows from the equivalence between symmetric trace‐free tensors and spherical harmonics, cf., e.g., appendix C of Novikov, Veraart, et al. (2018) $O\left(b^{2}\right)$, and has been used in a number of earlier works (Hansen et al. 2013; Jespersen et al. 2018; Lu et al. 2006). Hence, instead of performing an integral over a limited number of directions, we can calculate $\bar{W}$ very fast and exactly from the estimated W tensor by simply taking a full trace. This quantity is dimensionless (as is MK), and has qualitatively similar contrast to MK. The axial and radial kurtoses, AW and RW, are calculated as projections onto the principal fiber direction and onto the plane transverse to it, respectively:

(8) $$\begin{array}{c} \mathrm{AD}=D\left({\hat{v}}_{1}\right)\\ \mathrm{RD}=\int_{\left|\hat{n}\right|=1}\frac{d\hat{n}}{2\pi \;}D\left(\hat{n}\right)\delta \left(\hat{n}\cdot {\hat{v}}_{1}\right)\\ \mathrm{AW}=W\left({\hat{v}}_{1}\right)\\ \mathrm{RW}=\int_{\left|\hat{n}\right|=1}\frac{d\hat{n}}{2\pi \;}W\left(\hat{n}\right)\delta \left(\hat{n}\cdot {\hat{v}}_{1}\right) \end{array}$$

While MK, AK, and RK are more commonly used in literature than MW, AW, and RW, both parameter definitions will be generated in what follows, owing to the lower probability for outliers when using W compared with K (as shown in Figure S3).

### 2.5 Data Analysis and Statistics

To evaluate the effect size of the denoising step in preprocessing on any dMRI parameter *x* used in this study, we assess the variability across pairs of scans by the coefficient of variation ${\mathrm{CV}}_{x}=\sigma_{x}/\mu_{x}$, where the estimate of the mean is $\hat{\mu_{x}}=\frac{1}{2}\left(x_{1}+x_{2}\right)$, and the estimate of absolute error $\hat{\sigma_{x}}=\frac{\sqrt{\pi }}{2}\left|x_{1}-x_{2}\right|$. Here $x_{1,2}$ refer to the parameter estimates from the two scans being compared. Test–retest variability was compared within scanner, across scanners, and across echo times, on a region of interest (ROI) level, and using a voxel‐wise approach. In addition, concordance analysis and the concordance correlation coefficient (CCC) (Lawrence and Lin 1989) were used to assess reproducibility and agreement between scans.

*Voxel‐wise analysis*: For each subject, a multimodal template was generated based on *S*_0_ rotational invariant maps and FA maps from each of the five repeated acquisitions. The subject‐wise template was generated with Mrtrix3 (Tournier et al. 2019), using rigid registrations to a midpoint space, resulting in template maps for each of the 20 subjects. Next, a population template was generated based on all 20‐subject template *S*_0_ and FA pairs using nonlinear deformations (again using Mrtrix3). Rigid transformations from original space and warps to population space were concatenated, and then parametric maps for each subject, scan, and denoising level were transformed to the population template using cubic spline interpolation. For voxel‐wise analyses, CV were computed in the space of subject templates (after transforming parameters using rigid transforms) and for Figure 3 only, in the space of the unbiased population midpoint space (by transforming parameters using nonlinear transforms). Interpolation is applied during both rigid and nonlinear transformations, as cubic spline interpolation is applied when transforming parametric maps to the population template. However, the main distinction lies in the fact that nonlinear deformations introduce small additional distortions due to the nature of the warps, which may slightly confound interpretations in comparison to rigid transformations. We performed a concordance analysis showing the degree of concordance correlation between test–retest pairs in pooled voxels over all subjects and show Bland‐Altmann plots to measure the error between the same pairs.

*Harmonization comparison*: The effect of harmonization was compared to denoising for data with bias due to varying echo times (and noise floors). Harmonization was performed using the state‐of‐the‐art linear‐RISH (Mirzaalian et al. 2016) method. Linear‐RISH uses mean rotationally invariant spherical harmonic (RISH) features over a population to normalize those of each individual, and after normalization, comparisons were performed in subject's native space. All transformations were computed once and applied to all datasets to eliminate registration induced error. Denoising was performed on both magnitude and complex data (Figure 7).

We used the A‐1 (case 2) intraclass correlation coefficient (ICC; McGraw and Wong 1996) over all brain voxels to assess test–retest reliability in this study. This form of ICC measures the absolute agreement among measurements taken on randomly selected subjects, making it well‐suited for comparing results across different scanners and protocols where variability in acquisition settings is expected. The choice of ICC (A‐1) allows us to account for random effects and evaluate how consistently the same subjects' diffusion metrics are measured under varying conditions, ensuring that observed differences are not driven by systematic biases in hardware or protocols. We compared harmonization without denoising, denoising alone, and using both harmonization and denoising.

*ROI analyses* were performed in the original space of each scan (to minimize registration bias in CV measurements) by computing a nonlinear registration between the JHU white matter (WM) atlas (Hua et al. 2008) and the overall population template, and applying the inverse warps used to transform each subject map to the population average. Analyzed WM ROIs include genu, body, and splenium of the corpus callosum, internal capsule, and corona radiata. Since the original JHU ROIs (Hua et al. 2008) are quite large, we thresholded them in template space (not in subject space) by removing voxels in lowest 5th percentile of FA to minimize misregistration and partial‐volume effects. All these ROIs were combined into a single large WM region when tabulating full WM scale statistics. Gray matter (GM) ROIs were derived using Freesurfer (Desikan et al. 2006), and CVs were computed in the thalamus, as a representative gray matter region due to its key role in sensorimotor integration and its structural connections to various white matter tracts, which are critical in diffusion MRI studies. Additionally, the thalamus is a large central region and less prone to artifacts due to partial volume effects and EPI induced distortion. Freesurfer was performed using the MP‐RAGE acquired for each subject as an input, and ROIs were rigidly registered to diffusion space using the preprocessed b0 image as a registration reference. When taking ROI means, outliers in kurtosis metrics were thresholded prior to aggregating statistical metrics by excluding pixels with kurtosis < −1 or kurtosis > 10, and when quantifying the prevalence of outliers, the same range was used to determine the percentage of outlier voxels in a given white matter region (Figure S3).

*Noise floor analysis* was performed in diffusion weighted images to measure the level of bias reduction that comes from unwinding the denoised phase in complex data. We measured the noise floor by computing the spherical mean signal over directions 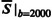 in the ventricles at *b* = 2000 s/mm (Jelescu and Budde 2017). Due to diffusion attenuation, there is negligible signal (∼*e* ^−6^≈0.002) left in CSF at this gradient strength, therefore 
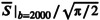
 can serve as a noise level estimator for a Rayleigh‐distributed random variable. Ventricular segmentation was performed using Freesurfer as described above. The noise floor was further normalized by the signal $S_{0}\equiv S{|}_{b=0}$ at *b* = 0:
(9)

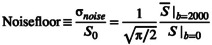




*Power analysis* was performed to test the number of subjects necessary to detect a 5% effect size based on group means and standard deviations observed in this healthy control population. A 5% effect size was chosen based on Cohen's *d* values reported in literature (Kochunov et al. 2022), where for various psychiatric disorders grou pwise differences in mean FA could range from 0% to 42%. To compute sample size, we used the formula:

(10) $$\begin{array}{c} n_{1}=\frac{2{\left(Z_{\alpha /2}+Z_{\beta }\right)}^{2}\sigma_{p}^{2}}{{\left(\mu_{x1}-\mu_{x2}\right)}^{2}} \end{array}$$

Here *n*_1_ is the sample size for a single group, *μ*_1,2_ refers to the means of the two groups being compared, we assume that the variance in measurements for the test and retest data are the same, and $\sigma_{p}^{2}$ is the sum of test–retest variance and intersubject variance,

(11) $$\begin{array}{c} {\sigma_{p}}^{2}=\text{var}\left(x_{1}\right)+{\bar{\sigma }}^{2} \end{array}$$

where $\bar{\sigma }$ is the population averaged $\sigma_{x}$ (true variance without the effect of measurement noise), and the variance of test measurements over the population (for *N* subjects) is $\mathrm{var}\left(x_{1}\right)=\frac{1}{N}\sum_{i=1}^{N-1}{\left(x_{i1}-\mu_{x1}\right)}^{2}$. *Z*_α_ is the *z* score corresponding to significance level *α*/2, and *Z*_β_ the critical value corresponding to the power of the test *β* (Kim 2016). This simulated the sample size required to reach voxel‐wise statistical significance in a two‐sided *t*‐test with 80% power and *α* = 0.05. We created two groups whose diffusion and kurtosis parameters differed by 5% and measured the number of subjects required to reach statistical significance. This analysis was performed for low‐SNR voxel‐wise data using test–retest CVs from Table 2 and high‐SNR ROI‐wise data using test–retest CVs from Table S1. For both comparisons intersubject variance was computed from ROI‐wise data and had a range from 0.03 to 0.04.

**TABLE 2:** Mean coefficients of variation (CV) for each DTI and DKI parameter and denoising type evaluated in four regions: Splenium of the corpus callosum, posterior limb of the internal capsule (PLIC), anterior corona radiata (ACR), and thalamus. We show CVs for each of the three comparisons: Within‐scan variability ($P_{92}^{\left(1\right)}$ vs. $P_{92}^{\left(2\right)}$ and $S_{127}^{\left(1\right)}$ vs. $S_{127}^{\left(2\right)}$), cross‐protocol variability ($P_{92}^{\left(1\right)}$ vs. $P_{127}^{\left(1\right)}$), and cross‐scanner variability ($P_{127}^{\left(1\right)}$ vs. $S_{127}^{\left(1\right)}$).

		PRISMA^(1)^_92_ PRISMA^(2)^_92_			SKYRA^(1)^_127_ SKYRA^(2)^_127_			PRISMA^(1)^_92_ PRISMA^(1)^_127_			PRISMA^(1)^_127_ SKYRA^(1)^_127_		
		Complex	Magnitude	None	Complex	Magnitude	None	Complex	Magnitude	None	Complex	Magnitude	None
SPLENIUM (CC)	MD	0.056	0.060	0.089	0.103	0.102	0.158	0.174	0.148	0.180	0.224	0.197	0.241
	AD	0.045	0.048	0.097	0.078	0.075	0.149	0.115	0.102	0.165	0.143	0.130	0.199
	RD	0.149	0.157	0.218	0.291	0.289	0.374	0.382	0.327	0.392	0.515	0.451	0.495
	FA	0.051	0.052	0.079	0.075	0.076	0.102	0.130	0.110	0.138	0.159	0.148	0.167
	MW	0.042	0.041	0.093	0.105	0.084	0.196	0.104	0.149	0.173	0.121	0.116	0.218
	AW	0.065	0.061	0.106	0.094	0.071	0.141	0.102	0.193	0.187	0.111	0.112	0.172
	RW	0.123	0.148	0.429	0.308	0.254	0.909	0.248	0.298	1.240	0.282	0.303	1.084
PLIC	MD	0.034	0.035	0.069	0.051	0.048	0.102	0.051	0.053	0.089	0.061	0.058	0.105
	AD	0.039	0.039	0.092	0.051	0.053	0.132	0.058	0.062	0.117	0.058	0.060	0.135
	RD	0.066	0.069	0.136	0.115	0.115	0.214	0.102	0.104	0.183	0.142	0.144	0.227
	FA	0.045	0.046	0.092	0.068	0.071	0.116	0.068	0.070	0.120	0.082	0.086	0.127
	MW	0.026	0.027	0.070	0.045	0.039	0.116	0.042	0.091	0.110	0.051	0.042	0.115
	AW	0.054	0.047	0.111	0.069	0.057	0.139	0.072	0.130	0.162	0.076	0.065	0.140
	RW	0.058	0.060	0.221	0.101	0.091	0.519	0.081	0.092	0.405	0.113	0.102	0.547
ACR	MD	0.039	0.041	0.074	0.069	0.067	0.121	0.063	0.064	0.100	0.079	0.074	0.127
	AD	0.045	0.045	0.098	0.072	0.065	0.145	0.072	0.071	0.126	0.076	0.070	0.147
	RD	0.062	0.064	0.112	0.107	0.104	0.177	0.089	0.091	0.162	0.122	0.116	0.191
	FA	0.081	0.083	0.152	0.116	0.115	0.198	0.113	0.109	0.223	0.130	0.130	0.197
	MW	0.031	0.029	0.074	0.065	0.050	0.128	0.058	0.113	0.133	0.070	0.059	0.131
	AW	0.053	0.045	0.107	0.081	0.058	0.136	0.079	0.158	0.167	0.089	0.064	0.141
	RW	0.059	0.057	0.188	0.115	0.092	0.421	0.103	0.086	0.364	0.119	0.100	0.450
Thalamus	MD	0.058	0.060	0.092	0.088	0.084	0.146	0.116	0.117	0.161	0.129	0.123	0.182
	AD	0.059	0.060	0.116	0.083	0.076	0.167	0.110	0.105	0.207	0.116	0.107	0.196
	RD	0.074	0.077	0.117	0.121	0.114	0.191	0.149	0.150	0.226	0.176	0.167	0.239
	FA	0.113	0.119	0.236	0.151	0.144	0.260	0.161	0.163	0.389	0.175	0.168	0.300
	MW	0.055	0.052	0.132	0.100	0.086	0.221	0.112	0.182	0.218	0.115	0.117	0.238
	AW	0.073	0.064	0.128	0.109	0.088	0.151	0.108	0.207	0.185	0.123	0.117	0.178
	RW	0.092	0.092	0.296	0.142	0.124	1.116	0.186	0.157	0.622	0.179	0.158	0.884

*Note:* The color shadings represent the minimum CV value (green), median CV (white), and maximum CV (red) over all comparisons.

## 3 Results

**FIGURE 2:**
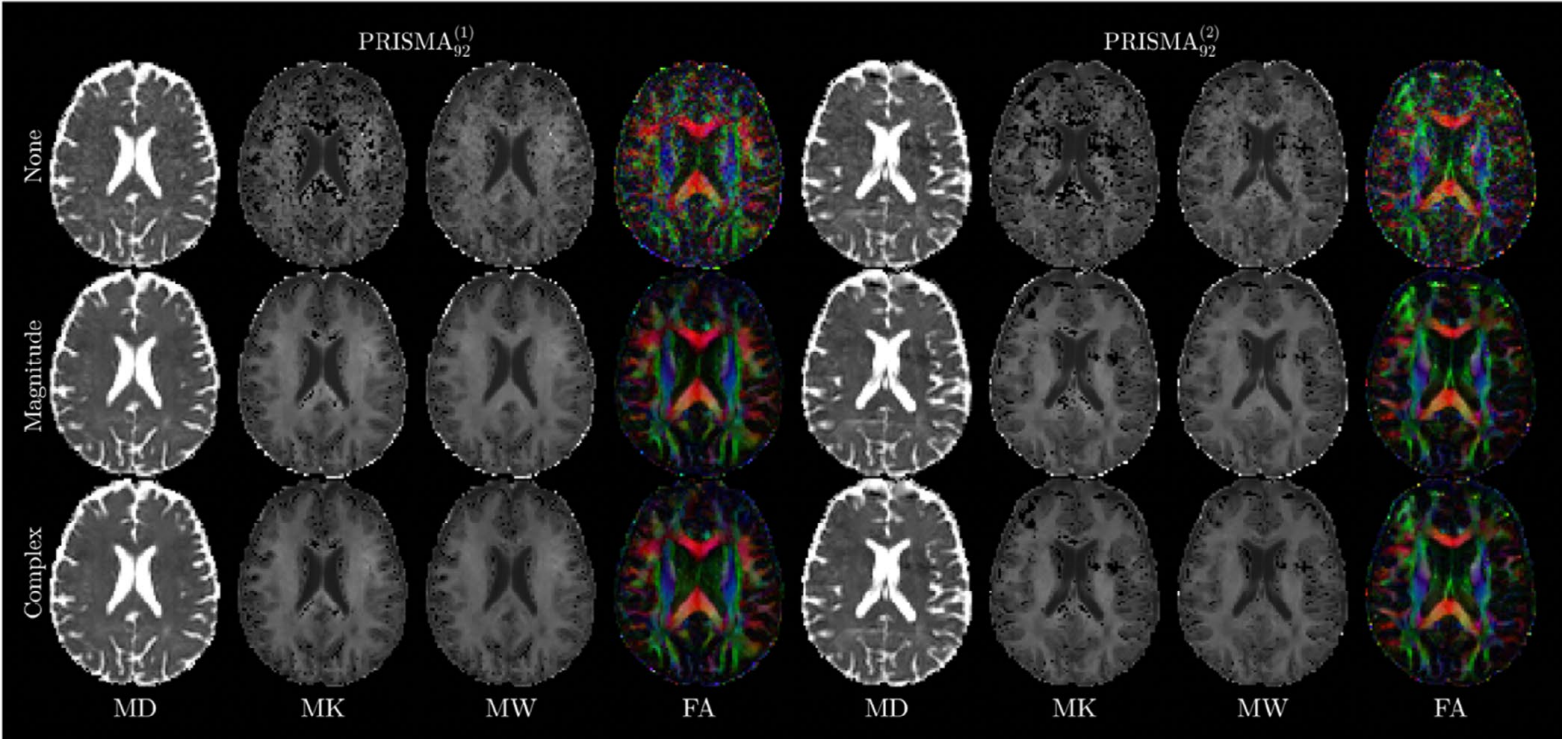
Illustration of different denoising methods on DTI and DKI measures (MD, MK, MW, and FA) across scan‐rescan data on the Prisma for a representative subject (26‐year‐old female). None: Non‐denoised data, Magnitude: MPPCA denoised magnitude data, Complex: MPPCA denoised complex data. Rician bias reduction is evident in the increased contrast of FA images in complex denoised data. Outliers were reduced in kurtosis maps both through denoising and the use of *W* representation.

Figure 2 shows representative diffusion and kurtosis maps for a single subject (26‐year‐old female) for test–retest data acquired on the Prisma scanner for MD, MK, MW, and FA. Two effects are notable from these maps: (1) The improved qualitative denoising effect from no denoising, to magnitude, to complex approaches. (2) The large decrease in kurtosis outliers observable as “black voxels” in MK maps both due to denoising, and by moving to the MW representation. This effect is also shown in Figure S3, where we show the reduction in outliers in white matter by switching from *K* to *W* representations, here the total percentage of outliers was reduced from over 70% on the Skyra system from MK to under 10% for MW.

### 3.1 Voxel‐Wise Variability

**FIGURE 3:**
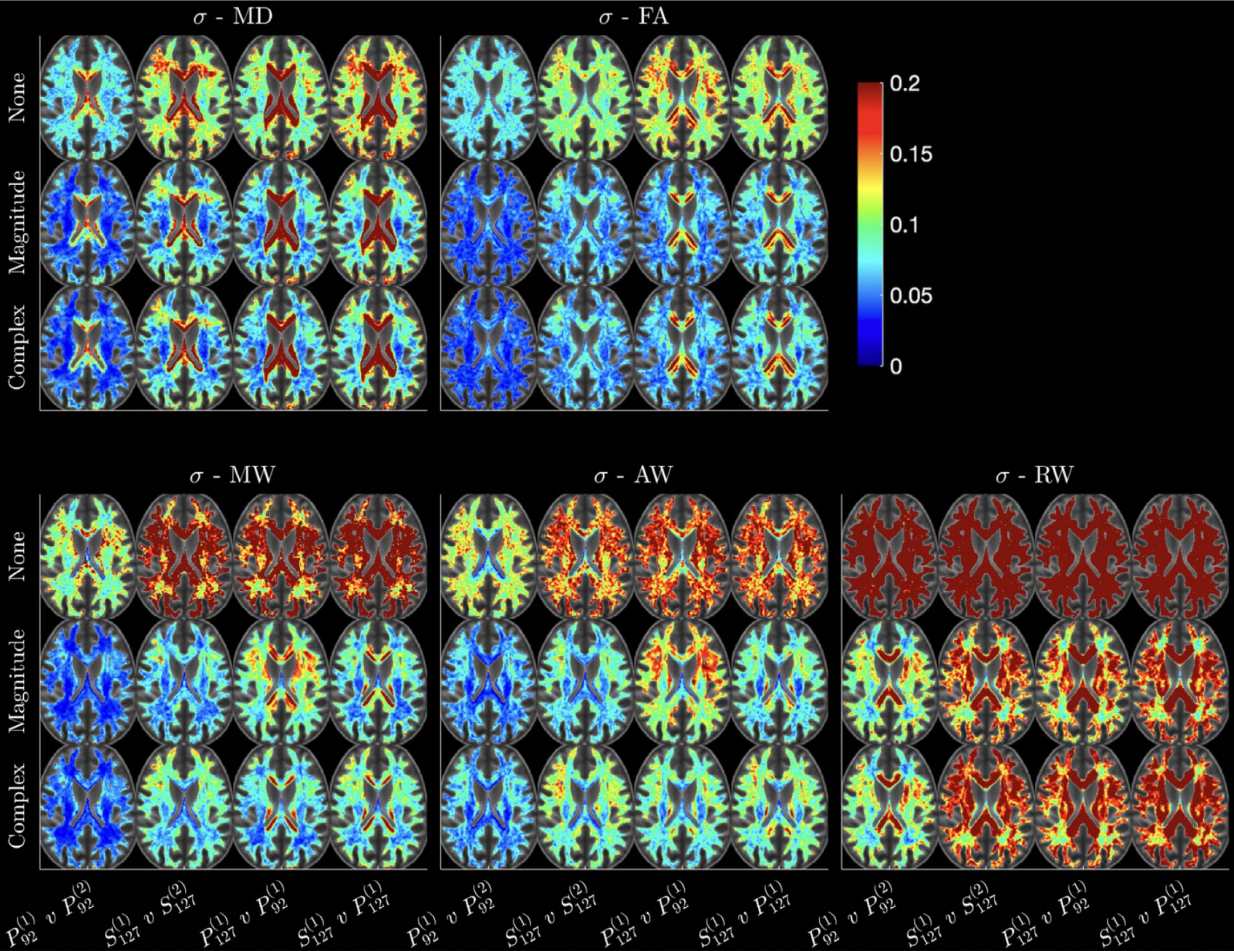
Maps of the absolute error $\sigma_{x}$, created by computing $\sigma_{x}$ in each subject's space, followed by warping into a common space and averaging over all 20 subjects. White matter masks were computed based on an FA threshold > 0.2 in the overall population template FA image. While interpolation affects both rigid and nonlinear transformations, the effect is more pronounced in the case of nonlinear warps, and thus, the error computed after nonlinear transformations are only used in this figure to avoid potential confounding in the interpretation of voxel‐wise results. For each parameter left two columns show within‐scan repeatability ($P_{92}^{\left(1\right)}$ vs. $P_{92}^{\left(2\right)}$ and $S_{127}^{\left(1\right)}$) vs. $S_{127}^{\left(2\right)}$), third column shows cross‐protocol variability ($P_{92}^{\left(1\right)}$ vs. $P_{127}^{\left(1\right)}$), and fourth column shows cross‐scanner variability ($P_{127}^{\left(1\right)}$ vs. $S_{127}^{\left(1\right)}$).

Voxel‐wise test–retest error maps averaged over subjects are shown in Figure 3 , for MD, FA, MW, AW, and RW (corresponding voxel‐wise maps for kurtosis are provided in Figure S1). Voxel‐wise CV is increased in regions near tissue boundaries because of noise limiting the precision of registration across subjects. However, this effect is reduced when using denoised dMRI, as visible in the MW‐map around the genu (Figure 3). We show $\sigma_{x}$ in Figure 3. rather than CV ($\sigma_{x}/\mu_{x}$) due to the potential instability when normalizing by small mean values. In regions where the mean ($\mu_{x}$) approaches zero, the CV can become disproportionately large, leading to misleading interpretations of variability. By focusing on $\sigma_{x}$, we provide a more stable and interpretable measure of dispersion that avoids these issues, particularly in low‐signal voxels.

To minimize the effect of misregistration, Table 2 lists the mean voxel‐wise CV pooled across different subjects within the splenium, posterior limb of the internal capsule (PLIC), anterior corona radiata (ACR), and thalamus, respectively. Regional CVs for kurtosis (K instead of W) including outliers are also shown in supplemental Figure S2. W‐parameters offer better robustness to outliers (see Figure S3), such that without denoising, MW in the internal capsule has voxel‐wise CV on the order of 9%–12% and denoising lowers test–retest variability on the Prisma system down to 3%–4%. FA has variability on the order of 7%–10% without denoising in highly aligned WM regions, and denoising lowers variability to 4%–6% for both Prisma and Skyra data.

The largest benefit of denoising and lowering the noise floor presents when comparing different echo times (Table 2). In AW we observe decreases in CV from 16.2% to 7.2% in the PLIC with complex denoising, along with corresponding large reductions in CV in other remaining WM areas. In line with the maps shown in Figure 3, we observe that CVs are consistently the lowest in areas such as the internal capsule, that are less prone to partial volume effects and Gibbs ringing. Indeed, regions near the ventricles such as the corpus callosum often have relatively higher variability.

**FIGURE 4:**
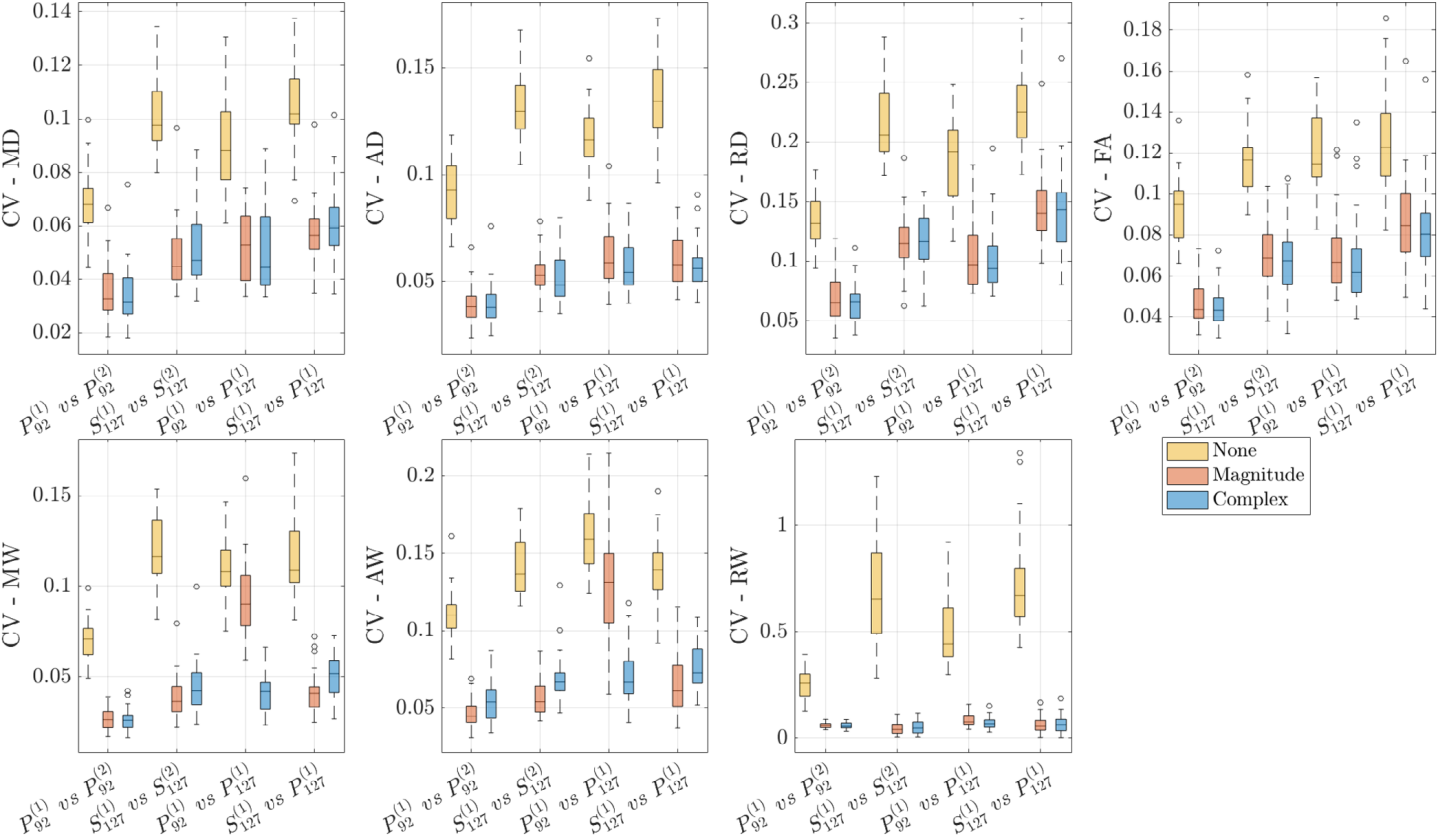
Box plots of voxelwise CV, $\sigma_{x}/\mu_{x}$, for each DTI and DKI parameter from pooling all voxels in whiter matter (PLIC and ALIC) over all 20 subjects. *X*‐axes show each of the four comparisons: Within‐scan variability ($P_{92}^{\left(1\right)}$ vs. $P_{92}^{\left(2\right)}$ and $S_{127}^{\left(1\right)}$ vs. $S_{127}^{\left(2\right)}$), cross‐protocol variability ($P_{92}^{\left(1\right)}$ vs. $P_{127}^{\left(1\right)}$), and cross‐scanner variability ($P_{127}^{\left(1\right)}$ vs. $S_{127}^{\left(1\right)}$). Note that CVs here are not subject to non‐linear warps, as they are pooled from subject‐wise templates rather than the population template.

Box plots of mean voxel‐wise CVs in PLIC are shown in Figure 4 over all subjects (corresponding boxplots for kurtosis CVs are provided in Supporting Information Material). We observe strongest effects of denoising in *W* between data with differing echo times ($P_{92}^{\left(1\right)}$ vs. $P_{127}^{\left(1\right)}$), where denoising complex data leads to a 12% decrease in CV in AW, along with marked improvements in CV in AD and FA from ∼9% down to 6%. Denoising (both magnitude and complex) give strong improvements in voxel‐wise CV in kurtosis parameters because denoising helps minimize outliers in both *K* and *W* maps. While complex and magnitude denoising often give very similar changes in CV, MP complex gives the greatest improvement in cross‐echo time reproducibility (3%–7% in MD, MW, and FA) because of the reduction in noise floor.

**FIGURE 5:**
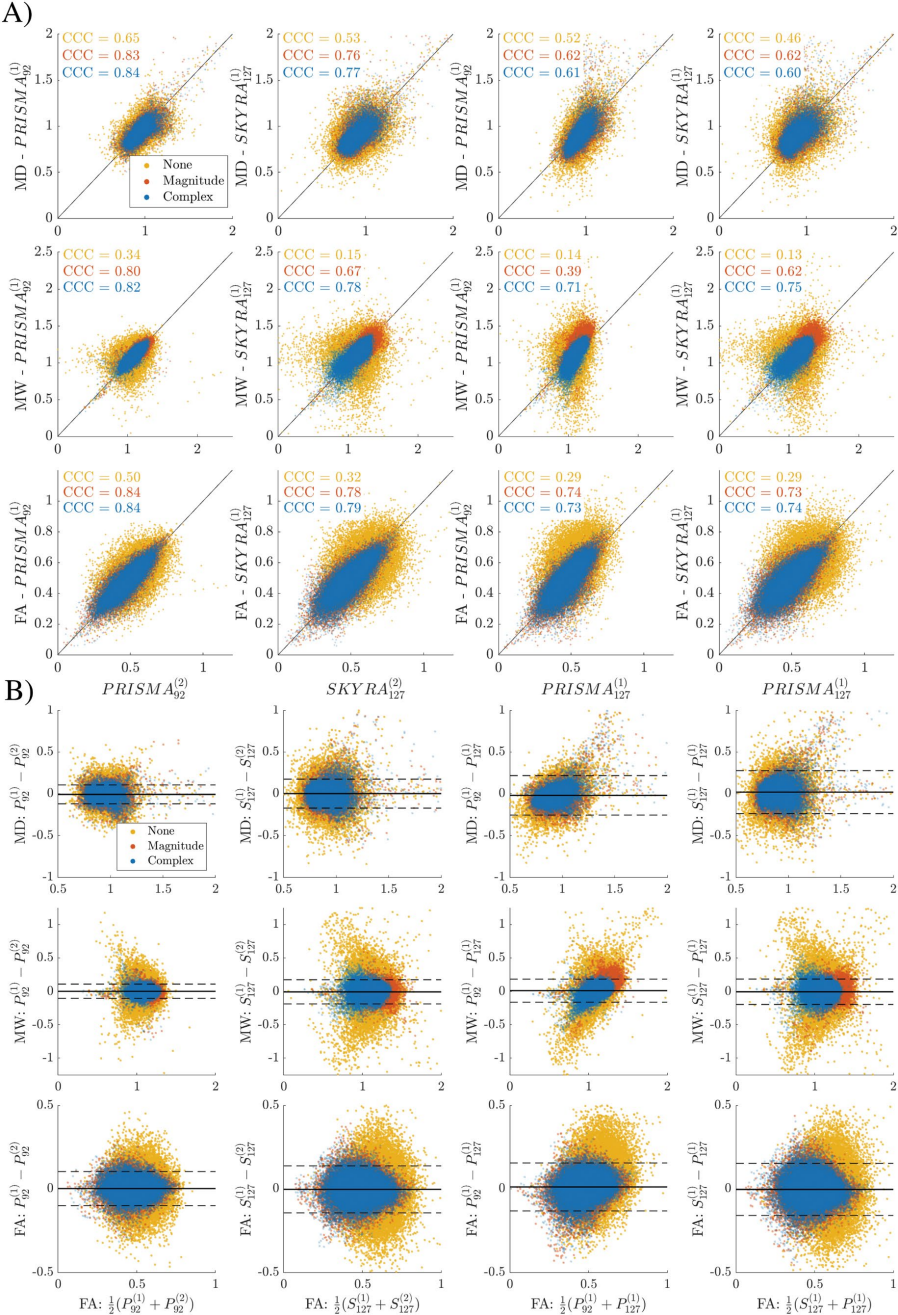
(a) Voxel‐wise scatter plots over white matter for all 20 subjects. Concordance correlation coefficients (CCC) are shown on the upper left corner of each plot for DTI and DKI parameters over white matter (internal capsule). (b) Bland–Altman plots over WM for all 20 subjects. *Y* axes show the error between test–retest measurements and *X* axes show the mean. Solid black line shows an error of 0, and dashed lines indicate error greater than 3 standard deviations away from the mean in complex denoised data.

Figure 5a,b show the results of concordance analysis on voxel‐wise data. It is visible from concordance correlation coefficients (shown in Figure 5b) that denoising leads to stronger correlations and lower variance in all test–retest datasets. In fact, without denoising, voxel‐wise correlations drop to as low as 0.13 across scanners and protocols in kurtosis values. Correlation and Bland–Altman analyses demonstrate the strongest correlations and narrowest voxel‐wise parameter distributions between scans on the same scanner with the same echo‐time after denoising. The correlations are lower for data acquired on different scanners (same echo time), or with different echo time (same scanner). Bland–Altman plots (Figure 5b) show the shift in parameter values resulting from decreased noise floor with complex denoising, particularly in MW. The middle row of Figure 5b shows how denoising complex data decreases WM kurtosis values while also minimizing test–retest error across all comparisons.

### 3.2 ROI‐Wise Variability

**FIGURE 6:**
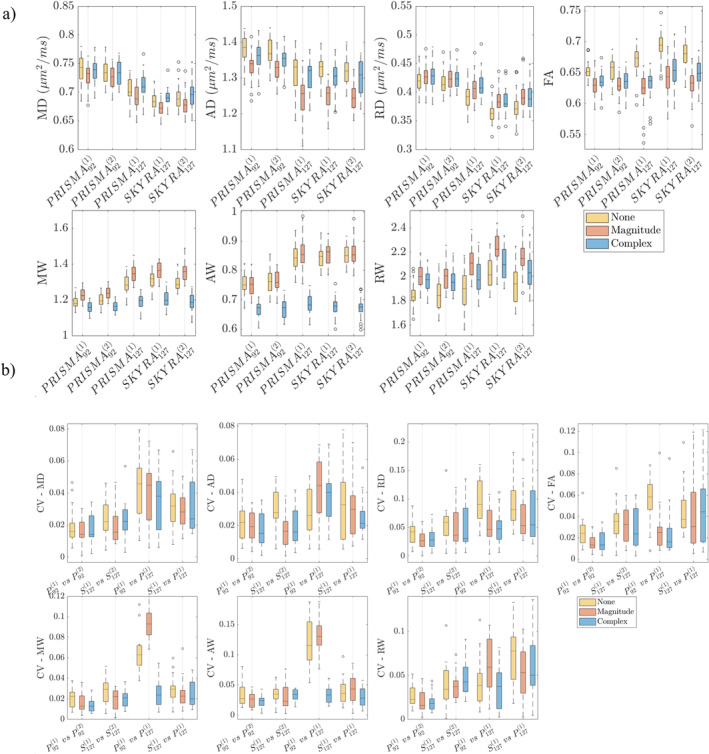
(a) Boxplots of average ROI‐values in white matter for each DTI and DKI parameters over all 20 subjects. (b) Boxplots of ROI‐wise CV for each DTI and DKI parameters over all 20 subjects, where $\sigma_{x}/\mu_{x}$, is from within the white matter ROI. The white matter ROI included PLIC, ALIC. DTI parameters are shown in the top row and DKI parameters in the bottom row. Box plots show each of the four comparisons: Cross‐protocol variability ($P_{92}^{\left(1\right)}$ vs. $P_{127}^{\left(1\right)}$), within‐scan variability ($P_{92}^{\left(1\right)}$ vs. $P_{92}^{\left(2\right)}$ and $S_{127}^{\left(1\right)}$ vs. $S_{127}^{\left(2\right)}$), and cross‐scanner variability ($P_{127}^{\left(1\right)}$ vs. $S_{127}^{\left(1\right)}$).

Figure 6a shows boxplots of ROI‐means DTI and DKI parameters, respectively, in WM over all 20 subjects, for all five scans, and for all denoising methods, and Figure 6b shows corresponding ROI‐wise CVs (rather than voxel‐wise CVs shown in Figure 4). DTI parameters were obtained from DTI‐fitting including *b* = 0 and *b* = 1000 s/mm^2^ (DTI parameters obtained from DKI‐fitting are shown in Figure S4). We observe variation in all parameter values according to TE. which can be related to corresponding SNR, which was derived as 13.1, 12.6, 9.3, 9.0, and 9.4 at *b* = 0‐image for $P_{92}^{\left(1\right)}$, $P_{92}^{\left(2\right)}$, $P_{127}^{\left(1\right)}$, $S_{127}^{\left(1\right)}$, and $S_{127}^{\left(2\right)}$, respectively. While magnitude denoising increased variability most pronouncedly in AD, both magnitude and complex denoising reduced the variability in FA, and complex denoising clearly reduced AW and MW, likely due to reduced effect of the noise floor.

There is less advantage to denoising when averaging over hundreds of voxels within a region as compared to voxel‐wise analyses (as SNR increases with the square root of the number of voxels in the ROI), but CVs still improve by 1%–9% (Table S1) for kurtosis parameters in the ACR when comparing data with differing echo times. We attribute this specific improved agreement in cross‐echo time data to the reduction in noise floor provided by complex denoising, which is more effective at mitigating noise‐related biases from longer echo times.

Figure 6b shows boxplots of ROI‐wise CV DTI parameters and DKI parameters, respectively in the PLIC over all 20 subjects, for the four comparisons, and for all three processing methods. Test–retest variability on Prisma and Skyra hover between 1% and 4% for diffusion and kurtosis values, and the lowest test–retest variability is observed on the Prisma due to its high SNR because of the shorter TE on this scanner. Cross‐protocol and cross‐scanner variability are higher due to inconsistency in echo time and hardware, respectively, resulting in greater differences in SNR between compared datasets. A notable decrease in CV is observed in AW and MW when examining cross‐protocol variability because of bias reduction through complex denoising—resulting in decreased eigenvalue repulsion and noise floor.

Table S1 shows mean values of ROI‐averaged CV over all 20 subjects. We found average test–retest CV for MD on the Prisma scanner to be about 1%–2% in WM, on the Skyra we found slightly larger CVs, but also on the order of 1%–2% regardless of whether denoising was used. Cross‐protocol CV was higher, in the regime of 3%–4%, and cross‐scan CV in the range of 2%–3%. Complex denoising consistently lowers variability in diffusivity, however this benefit was quite small (0.5%–1.0% improvements) at the level of the ROI. The strongest reduction in variability is observed for AW and complex denoising thanks to noise floor reduction prior to ROI averaging.

### 3.3 Comparison Between Harmonization and Denoising

**FIGURE 7:**
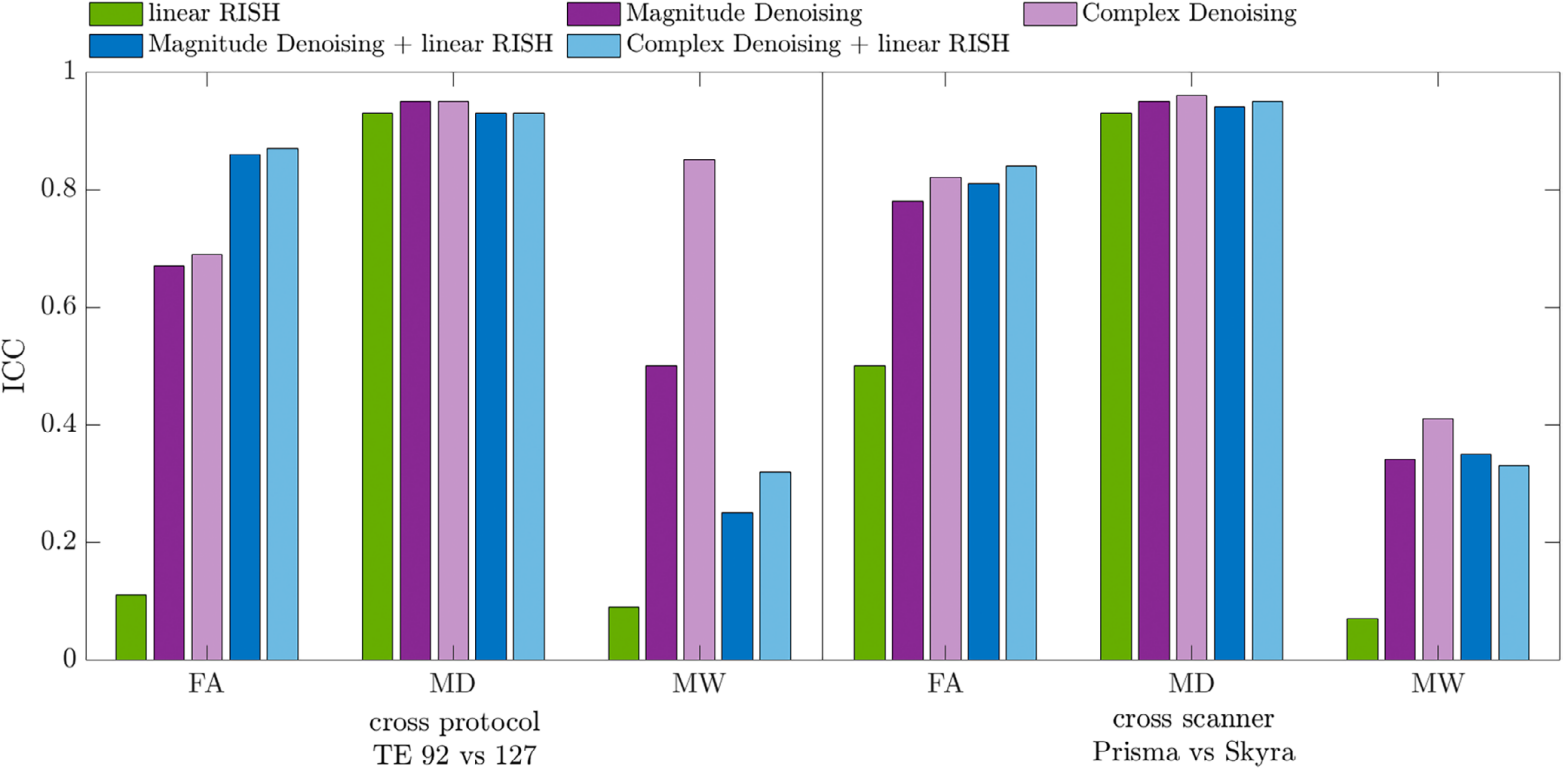
Intraclass correlation coefficients for two comparisons: Cross‐protocol variability, and cross‐scanner variability. Denoising of both complex and magnitude data consistently improves the ICC.

RISH harmonization was applied to voxel‐wise data. Figure 7 shows ICCs across protocol or across scanner for data processed with magnitude or complex denoising and/or harmonization. ICC based on harmonization alone is lowest for FA and MW, both metrics that are heavily affected by noise propagation. MD, which is more robust to noise, has high ICC both with harmonization alone and with denoising alone. In both cross‐protocol and cross‐scanner comparisons MW had the greatest benefit from combining denoising with linear‐RISH, where cross‐protocol, magnitude denoising improved the ICC from 0.09 to 0.32, and magnitude denoising+RISH further improved the ICC to 0.85. The same trend was observed in the cross‐scanner comparison over MW, but to a lesser extent. In both comparisons, FA showed a decrease in ICC with denoising+RISH compared to denoising alone.

### 3.4 Noise Floor Estimation

**FIGURE 8:**
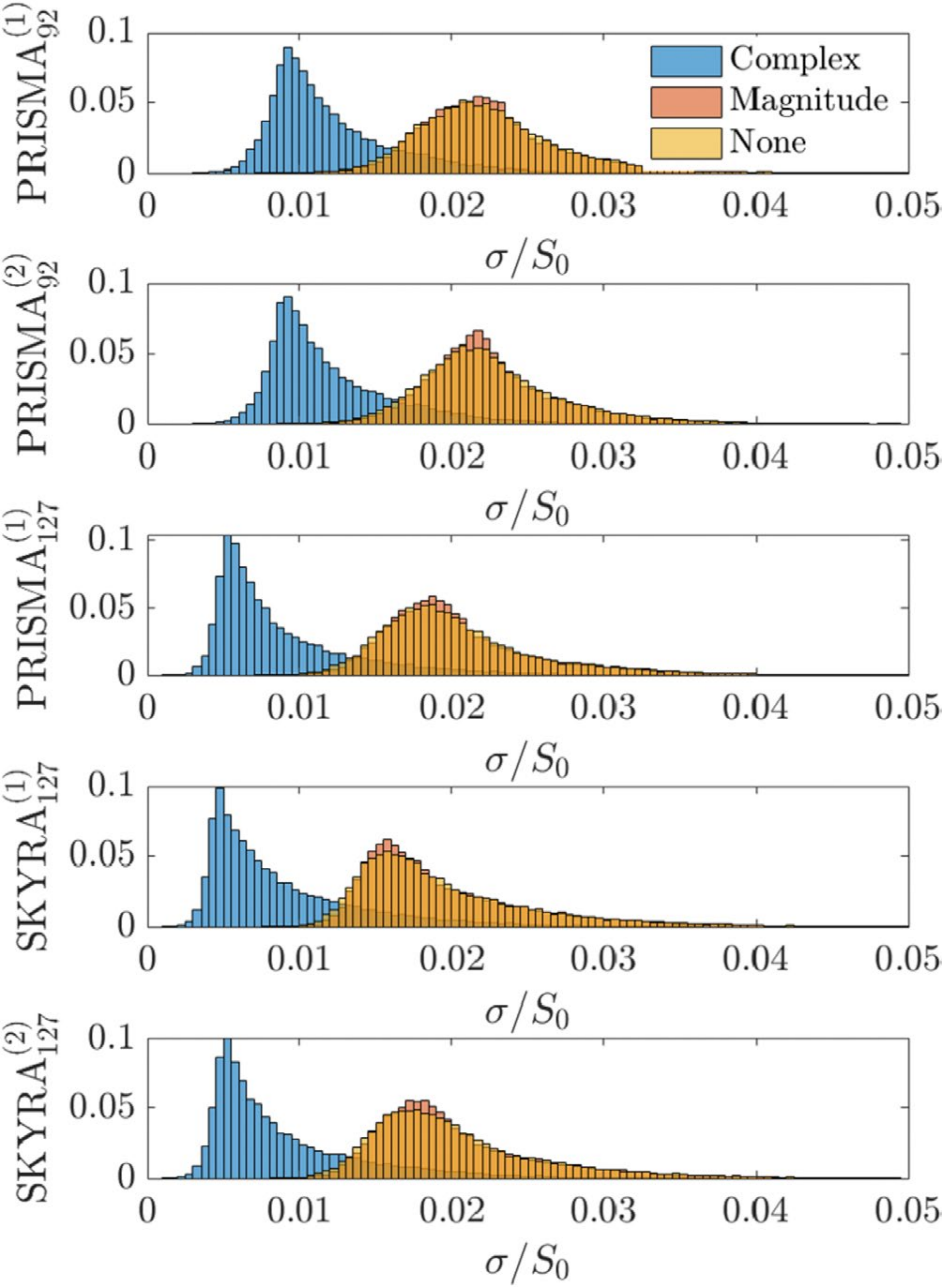
Noise floor estimation for each of the five scans according to Equation (9). Distributions shown come from pooled voxels in the ventricles over all subjects. As expected, MP magnitude shows the same noise floor as no‐denoising (as it does not remove the Rician bias). MP complex decreases the noise floor by a factor of 2–4.

Figure 8 shows the noise floors for dMRI data denoised using MP‐complex, MP‐magnitude, and no denoising for each of the five scans. We found that the baseline noise level for non‐denoised (or magnitude denoised) data is about 1.8%–2.5% of the dynamic range of the DWI dataset. Complex denoising lowers the noise floor to 0.4%–1%, on the Prisma system constituted a 2.5× reduction in noise floor. This reduction in noise floor propagates through tensor estimation and leads to the decreases in parameter bias present in Figure 6a. We note that the noise floor is higher in the low echo‐time Prisma data compared to the data acquired with TE = 127 ms, which may be attributable to contributions from physiological noise or increased B0 inhomogeneities present in lower TE data, even though this data has higher overall SNR.

### 3.5 Statistical Power Estimation

**FIGURE 9:**
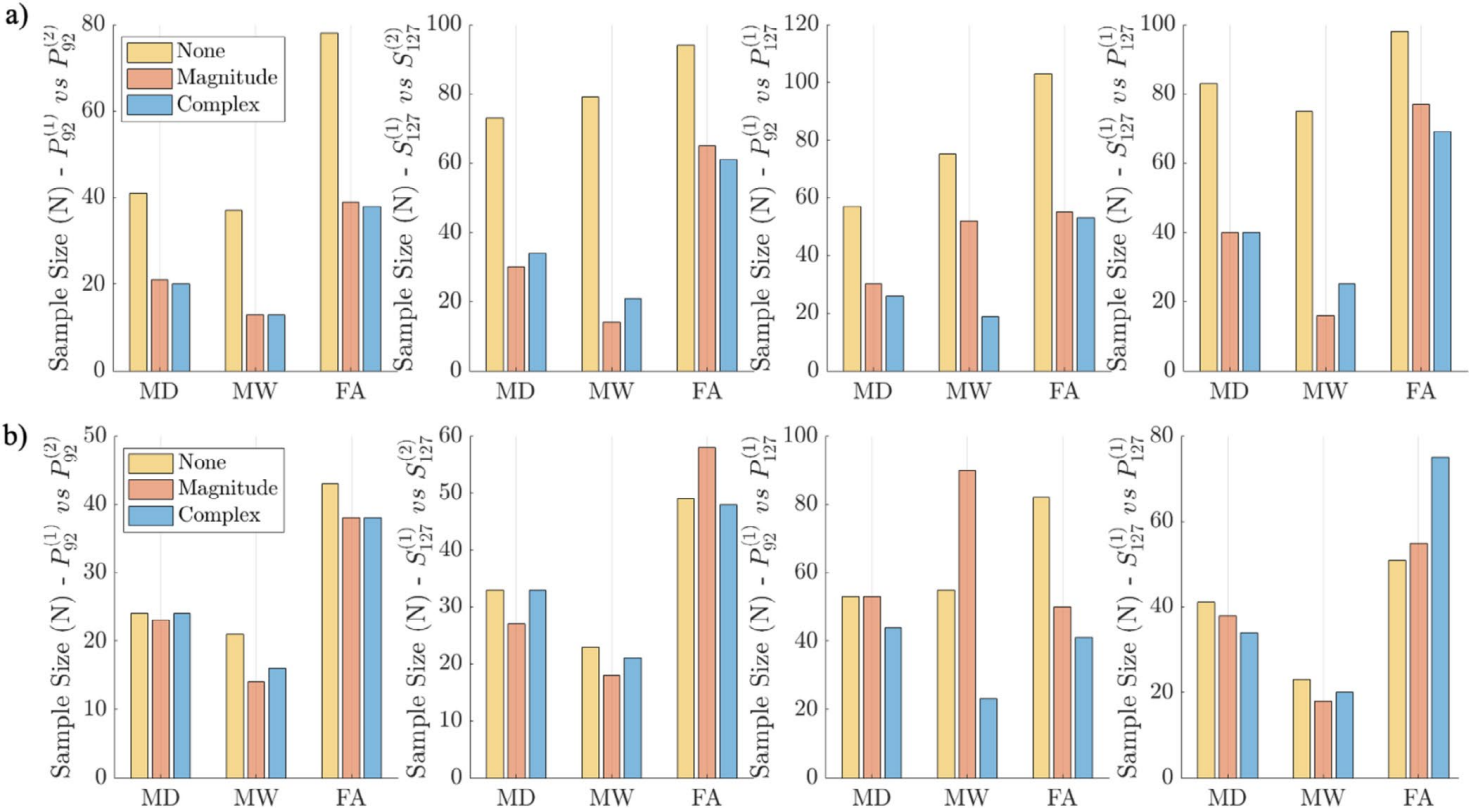
(a) Required sample size needed to significantly distinguish groups separated by an effect size of 5% with *α* = 0.05 and a power of 0.8 when performing voxel‐wise analysis, based on parameter ROI means and voxel‐wise test–retest standard deviations over all 20 subjects. (b) Required sample size needed when performing ROI‐based analysis, based on parameter ROI means and ROI‐wise test–retest standard deviations. Rows show sample sizes needed for four scenarios, with either cross‐scanner variability ($P_{92}^{\left(1\right)}$ vs. $P_{92}^{\left(2\right)}$ and $S_{127}^{\left(1\right)}$ vs. $S_{127}^{\left(2\right)}$), cross‐protocol variability ($P_{92}^{\left(1\right)}$ vs. $P_{127}^{\left(1\right)}$), and cross‐scanner variability ($P_{127}^{\left(1\right)}$ vs. $S_{127}^{\left(1\right)}$).

Figure 9 shows the results of a statistical estimate of the sample size required to detect 5% difference in group means, for groups with equal variance. This analysis was performed for a voxel located in a common WM region (PLIC). Here we found that denoising universally lowers the number of subjects required to reach statistical significance (*p* = 0.05 in a two‐sided *t*‐test). For voxel‐wise data (Figure 9a), on the Prisma scanner, denoising allowed for sample size decreases of 51.2%, 64.9%, and 50% for MD, MW, and FA, respectively. On the Skyra we found that denoising allowed for corresponding decreases of 53.4%, 73.4%, and 35.9%. Both magnitude and complex denoising reduced the required sample size from 40–80 subjects (MD and MW) down to 20–40 subjects for MD and 10–40 subjects for MW, and from 70–100 subjects down to 40–70 subjects for FA. Both complex and magnitude denoising produced similar effects, with the notable exception that when comparing groups who underwent scans with differing TE, where for MW, complex denoising provided an addition 66.6% reduction in sample size compared to magnitude denoising. For ROI‐wise data (Figure 9b), the high SNR enabled by ROI averaging led to minimal sample size changes between denoised and non‐denoised data.

For data acquired using protocols with different TE ($P_{92}^{\left(1\right)}$ vs. $P_{127}^{\left(1\right)}$), complex denoising had the largest impact on sample size improvement because it offers a correction for differing noise floors. Complex denoising allowed for a sample size decrease of 53.3%, 73.9%, and 60% in MD, MW, and FA. On different scanners with the same TE ($S_{127}^{\left(1\right)}$ vs. $P_{127}^{\left(1\right)}$), both magnitude and complex denoising allows for a decrease in sample size of similar magnitude, hence we can conclude that these datasets contain similar noise floors.

## 4 Discussion

The goal of this study was to measure the reproducibility of higher‐order diffusion MR metrics. Denoising (Veraart, Fieremans, and Novikov 2016) of magnitude or complex diffusion MRI data, together with targeted artifact removal (Ades‐Aron et al. 2018; Chen et al. 2024), improves reproducibility of higher‐order diffusion parameters across scanners and protocols. Denoising reduces variations across test–retest datasets from ∼15%–20% to ∼5%–10% in kurtosis indices at the level of individual voxels. Subsequent measurements on different scanners and different TE (92 ms, 127 ms) were found to have voxel‐wise precision varying from 3% to 15% for both DTI and DKI metrics after denoising. Notably, we found the greatest improvement in reproducibility across data with differing signal‐to‐noise ratios (from varying echo times) when applying MPPCA to complex‐valued data, likely because of the ∼2.5× noise floor reduction offered by complex denoising.

When comparing denoising directly to harmonization using linear‐RISH, denoising outperformed harmonization in reducing bias from varying noise floors. Moreover, combining denoising with harmonization in voxel‐wise assessments improved intra‐scanner test–retest ICCs by 55% for FA, over harmonization alone. This highlights denoising's critical role in maximizing data precision and reliability in multi‐site dMRI studies.

This works stands out compared to previous works due to the comprehensive nature of the multi‐shell diffusion MRI dataset of 20 subjects, which allows for evaluating three separate types of reproducibility, along with analysis of how noise varies in repeated voxel‐wise measurements. Previous studies aimed to evaluate harmonization schemes include denoising as an aspect of their harmonization (Ning et al. 2020) either directly, or because of neural network harmonization where denoising is implicit to the network due to clean supervised training data. The values reported here are largely in line with prior results of DTI repeatability found in the literature (Kristo et al. 2013; Laguna et al. 2020; Liu et al. 2014; Vollmar et al. 2010) where for ROIs in the corpus callosum, FA has been shown to have ∼2%–3% within‐scan and between‐scan variability in aligned WM regions. Our reported values also agree with a previous report of intra‐scanner ROI mean kurtosis repeatability of 1%–4% (Kamiya et al. 2020). Similarly, we also show correspondence with reported reproducibility (Coelho et al. 2022) for tissue microstructure parameters. Such reproducibility may improve our ability to measure subtle changes in nervous tissue functional organization and complexity.

For this work, we employed an established though perhaps less commonly used definition for kurtosis (Hansen et al. 2013; Jespersen et al. 2018; Lu et al. 2006), (Equation 7), as we observed that kurtosis maps using the traditional definition, (Equation 6) feature more outliers due to unphysical kurtosis values or “black voxels” (Figure 2) as compared to the alternative definition (Equation 7), as also quantified in Figure S3. Alternatively, image correction methods, for example, based on the MK‐curve (Zhang et al. 2019), can also reduce the occurrence of “black voxels” and thereby improve the stability of kurtosis estimates, and subsequently increase the robustness and reproducibility of DKI measurements (Christiaanse et al. 2023). Such correction methods are complementary to our proposed denoising methods and could be combined for optimal robustness and reproducibility.

We found that MPPCA denoising has a strong impact on voxel‐wise data, lowering CVs in kurtosis from ∼40% down to 5%. As shown in Figure 4, the effect is most noticeable in homogeneous brain regions. However, voxel‐wise CV is nonuniform across WM due to different levels of tissue homogeneity in different regions, and CV is typically higher in regions with tissue boundaries such as the genu and splenium of the corpus callosum, owing largely to the effect of registration‐based spatial interpolations along with small misregistration effects. Similarly, voxel‐wise CV in GM (thalami) is higher than in homogenous WM owing to heterogeneous tissue composition.

There is less of a need for denoising when analyzing large groups of voxels (Figure 6b and Table S1). Indeed, ROI analyses are more reproducible because of averaging many voxels compared to voxel‐wise analyses (Kornaropoulos et al. 2022), but may be also less specific and obscured due to spatially local effects. Conversely, voxel‐wise analyses better capture biological variability, in which case denoising profoundly improves reproducibility. Indeed, voxel‐wise, cross‐protocol, and cross‐scanner CVs for non‐denoised diffusion parameters reach up to 20%, and after denoising variability drops to 3%–8% in most areas (Table 2). DTI differences between groups for varying psychiatric disorders can be as low as 1% and up to 50% effect size (Hansen et al. 2013), therefore our observed reduction in CV (Figure 4) gained through denoising may be clinically useful.

In line with the observed reduction in voxel‐wise CV after denoising, we also found that denoising decreases the sample size needed to reach statistical significance between groups separated by a 5% effect size by about 50% in MD, MW, and FA when performing a voxel‐wise analysis (Figure 9a). The degree of sample size reduction is determined by the underlying SNR of the dataset. In the voxel‐wise data, the intrinsic low SNR (SNR = 13 at TE = 92 ms and SNR = 9 at TE 127) of our diffusion protocol (due to small voxel size, short scan time, and minimal reconstruction), led to the most pronounced sample size reductions after denoising.

Conversely, and in line with results in Figure 6b, after increasing the SNR through ROI averaging, denoising had minimal (5%) to no impact on the required sample size for denoising (Figure 9b). At the level of ROIs 24 patients are required to detect a 5% difference in mean MD using a 2 sided t‐test regardless of denoising strategy, which is in‐line with predicted sample sizes from literature (Laguna et al. 2020; Szczepankiewicz et al. 2013).

Figure 6a shows the effect of varying TE, and corresponding SNR, on DTI and DKI parameters. While there is no ground truth for those in vivo values, we know that repulsion due to noise causes artificial increases to FA, AD, and decreases in RD (Basser and Pajevic 2000; Sprenger et al. 2016), an effect that is SNR dependent, and is reduced after denoising. Furthermore, the eigenvalue shrinkage step during denoising has been shown (Chen et al. 2024) to bias DTI‐parameters, another SNR‐dependent effect that is induced by denoising, with decreased AD values (Chen et al. 2024) by −4% at SNR = 10. Finally, the effect of bias due to noise floor is also SNR‐dependent, and can be reduced by complex denoising. All these effects may impact the outcome DTI/DKI parameters, as illustrated in Figure 6b, where shrinkage may explain the increased variability in AD after magnitude denoising, and reduced eigenvalue repulsion may explain the reduced variability in FA after both magnitude and complex denoising. In addition, complex denoising reduced the effect of the noise floor, resulting in lower and more uniform AW and MW across TE.

The additional benefit of MPPCA denoising of complex dMRI data (magnitude and phase) compared to denoising magnitude dMRI data is most pronounced in reducing parameter bias of higher‐order dMRI metrics such as AW and to a lesser effect MW, as shown in Figure 4 and Figure 5a, particularly lowering the variability between protocols with different TE. Interestingly, the deviation of the regression slope from 1 in cross echo time (92 vs. 127 ms) CV of MW data (Figure 5b) is reduced most after complex denoising due to lowering the noise floor caused by higher noise floor in the long‐TE scan.

Complex MP‐denoising has the potential for further improvement since the algorithm uses a “two‐pass” process, where we run the MP algorithm twice, first to estimate the noise free signal phase and then to perform the actual denoising. For this method to work effectively, these two steps must be sufficiently independent, that is, the first step should not introduce noise correlations into the second step. If the patches used in each step are too similar, the denoising will not perform as robustly in the second denoising step. In this study we tried to make the patches as different as possible (15 × 15 2D patches during phase estimation, 3D adaptive patches during the actual denoising), since the noise correlations introduced by the phase unwinding in the first step should be made as small as possible to maximize the performance of this approach. This is the reason why in some cases ($P_{127}^{\left(1\right)}$ vs. $S_{127}^{\left(1\right)}$ AW in Figure 5a) we observe slightly better denoising performance in MP‐magnitude compared with MP‐complex.

Here we show that the MPPCA denoising approach provides not only a notable and quantifiable improvement in reproducibility of cross‐protocol dMRI parameters, but also provides a powerful tool for data harmonization (by means of reducing the noise floor). Indeed, due to the different echo times and numbers of gradient directions used in $P_{92}^{\left(1\right)}$ and $P_{127}^{\left(1\right)}$ protocols, the resulting dMRI data have differing SNR and noise floor (see Figure 8). Without denoising, this results in parametric maps with differing levels of bias, and different numbers of outliers in kurtosis parameters owing to the different levels of precision in the raw data. Hence, reducing the noise floor by denoising complex dMRI data has the potential to improve parameter accuracy and *harmonize* data from sources with different SNR and thus, different noise floor. This is exemplified by the results of the harmonization analysis (Figure 7). We found that the impact of both complex denoising and harmonization was greatest when adjusting for the noise floor induced bias in FA in data with differing echo times. Since complex denoising can accurately reduce noise floor bias, it should be included as an essential first step to harmonizing data from separate sources.

Limitations of this study include the lack of a ground truth inhibiting us from knowing the exact degree of bias and variance induced by protocol specific effects. Recent work (Chen et al. 2024) showed that eigenvalue shrinkage can bias DTI and DKI parameters, particularly at low SNR, illustrated in Figure 6a, which warrants further investigation into its usefulness when combining dMRI with different noise levels. In addition, there are also additional sources of test–retest variability that were not measured here, including differing vendors, field strengths, *q*‐space sampling regimes, and number of head coils, but could be the subject of future work. The custom reconstruction pipeline used in this study allowed for saving of both magnitude and phase images, which facilitated both magnitude and complex denoising. When using vendor and sequence‐specific online reconstruction pipelines, phase map preservation, needed for complex denoising, may not be as readily accessible, and potential unknown reconstruction steps (e.g., filtering, interpolation) may interfere with the MPPCA denoising algorithm. Nevertheless, the core principles of our analysis, particularly the use of complex data for denoising, remain applicable and can be adapted to various reconstruction pipelines. We assessed only young adult healthy controls, and it should be noted that age or pathological changes to tissue microstructure may also increase diffusion parameter variability.

MP complex code is available as part of the DESIGNER‐v2 diffusion MRI processing pipeline (https://github.com/NYU‐DiffusionMRI/DESIGNER‐v2) and can be run either through python or through a dedicated Docker container.

## 5 Conclusion

MPPCA denoising reduces variability across scanners and echo times to 5%–10% voxel‐wise and 3%–5% at the ROI‐level for DTI and DKI metrics and has the potential to minimize the sample size required for voxel‐wise population‐wise statistics by 40%–70% depending on SNR. Denoising of complex dMRI enables noise floor reduction by up to 60%. Such improvement—that measurements from *individual voxels* become quantitative and reproducible—is an essential step towards bringing quantitative MRI and tissue microstructure imaging with MRI to clinical practice.

## Conflicts of Interest

The authors, including B.A.A., G.L., T.M.S., D.S.N., E.F., and N.Y.U. School of Medicine, hold stock in Microstructure Imaging Inc., which develops post‐processing tools for advanced MRI methods. Additionally, D.S.N., E.F., and J.V. are co‐inventors of a patent co‐owned by the University of Antwerp and NYU School of Medicine, describing technology evaluated in this study.

## Supporting information


**Data S1.** Supporting Information.

## Data Availability

The data that support the findings of this study are available from the corresponding author upon reasonable request.

## References

[hbm70142-bib-0001] Ades‐Aron, B. , J. Veraart , P. Kochunov , et al. 2018. “Evaluation of the Accuracy and Precision of the Diffusion Parameter EStImation With Gibbs and NoisE Removal Pipeline.” NeuroImage 183: 532–543. 30077743 10.1016/j.neuroimage.2018.07.066 PMC6371781

[hbm70142-bib-0002] Ahn, C. , and Z. Cho . 1987. “A New Phase Correction Method in NMR Imaging Based on Autocorrelation and Histogram Analysis.” IEEE Transactions on Medical Imaging 6, no. 1: 32–36. 18230424 10.1109/TMI.1987.4307795

[hbm70142-bib-0003] Aja‐Fernández, S. , A. Tristán‐Vega , and C. Alberola‐López . 2009. “Noise Estimation in Single‐ and Multiple‐Coil Magnetic Resonance Data Based on Statistical Models.” Magnetic Resonance Imaging 27, no. 10: 1397–1409. 19570640 10.1016/j.mri.2009.05.025

[hbm70142-bib-0004] Alexander, D. C. , T. B. Dyrby , M. Nilsson , and H. Zhang . 2019. “Imaging Brain Microstructure With Diffusion MRI: Practicality and Applications.” NMR in Biomedicine 32, no. 4: e3841. 29193413 10.1002/nbm.3841

[hbm70142-bib-0005] Amartur, S. , and E. M. Haacke . 1991. “Modified Iterative Model Based on Data Extrapolation Method to Reduce Gibbs Ringing.” Journal of Magnetic Resonance Imaging 1, no. 3: 307–317. 1802144 10.1002/jmri.1880010309

[hbm70142-bib-0007] Andersson, J. L. , and S. N. Sotiropoulos . 2016. “An Integrated Approach to Correction for Off‐Resonance Effects and Subject Movement in Diffusion MR Imaging.” NeuroImage 125: 1063–1078. 26481672 10.1016/j.neuroimage.2015.10.019 PMC4692656

[hbm70142-bib-0006] Andersson, J. L. , M. S. Graham , E. Zsoldos , and S. N. Sotiropoulos . 2016. “Incorporating Outlier Detection and Replacement Into a Non‐Parametric Framework for Movement and Distortion Correction of Diffusion MR Images.” NeuroImage 141: 556–572. 27393418 10.1016/j.neuroimage.2016.06.058

[hbm70142-bib-0008] Bao, L. , M. Robini , W. Liu , and Y. Zhu . 2013. “Structure‐Adaptive Sparse Denoising for Diffusion‐Tensor MRI.” Medical Image Analysis 17, no. 4: 442–457. 23541286 10.1016/j.media.2013.01.006

[hbm70142-bib-0009] Basser, P. J. , and S. Pajevic . 2000. “Statistical Artifacts in Diffusion Tensor MRI (DT‐MRI) Caused by Background Noise.” Magnetic Resonance in Medicine 44, no. 1: 41–50. 10893520 10.1002/1522-2594(200007)44:1<41::aid-mrm8>3.0.co;2-o

[hbm70142-bib-0010] Beck, D. , A.‐M. G. de Lange , I. I. Maximov , et al. 2021. “White Matter Microstructure Across the Adult Lifespan: A Mixed Longitudinal and Cross‐Sectional Study Using Advanced Diffusion Models and Brain‐Age Prediction.” NeuroImage 224: 117441. 33039618 10.1016/j.neuroimage.2020.117441

[hbm70142-bib-0011] Bernstein, M. A. , K. F. King , and X. J. Zhou . 2004. Handbook of MRI Pulse Sequences. Amsterdam; Boston: Elsevier Academic Press.

[hbm70142-bib-0012] Breuer, F. A. , M. Blaimer , R. M. Heidemann , M. F. Mueller , M. A. Griswold , and P. M. Jakob . 2005. “Controlled Aliasing in Parallel Imaging Results in Higher Acceleration (CAIPIRINHA) for Multi‐Slice Imaging.” Magnetic Resonance in Medicine: An Official Journal of the International Society for Magnetic Resonance in Medicine 53, no. 3: 684–691. 10.1002/mrm.20401 15723404

[hbm70142-bib-0013] Brey, W. W. , and P. A. Narayana . 1988. “Correction for Intensity Falloff in Surface Coil Magnetic Resonance Imaging.” Medical Physics 15, no. 2: 241–245. 3386597 10.1118/1.596255

[hbm70142-bib-0014] Buades, A. , B. Coll , and J.‐M. Morel . 2005. “A Review of Image Denoising Algorithms, With a New One.” Multiscale Modeling and Simulation 4, no. 2: 490–530.

[hbm70142-bib-0015] Chen, J. , B. Ades‐Aron , H.‐H. Lee , et al. 2024. “Optimization and Validation of the DESIGNER Preprocessing Pipeline for Clinical Diffusion MRI in White Matter Aging.” Imaging Neuroscience 2: 1–17. 10.1162/imag_a_00125 PMC12247605 40800272

[hbm70142-bib-0016] Christiaanse, E. , P. O. Wyss , A. Scheel‐Sailer , et al. 2023. “Mean Kurtosis‐Curve (MK‐Curve) Correction Improves the Test–Retest Reproducibility of Diffusion Kurtosis Imaging at 3 T.” NMR in Biomedicine 36, no. 3: e4856. 36285630 10.1002/nbm.4856 PMC10078439

[hbm70142-bib-0017] Coelho, S. , S. H. Baete , G. Lemberskiy , et al. 2022. “Reproducibility of the Standard Model of Diffusion in White Matter on Clinical MRI Systems.” NeuroImage 257: 119290. 35545197 10.1016/j.neuroimage.2022.119290 PMC9248353

[hbm70142-bib-0018] Cordero‐Grande, L. , D. Christiaens , J. Hutter , A. N. Price , and J. V. Hajnal . 2019. “Complex Diffusion‐Weighted Image Estimation via Matrix Recovery Under General Noise Models.” NeuroImage 200: 391–404. 31226495 10.1016/j.neuroimage.2019.06.039 PMC6711461

[hbm70142-bib-0019] Cox, S. R. , S. J. Ritchie , E. M. Tucker‐Drob , et al. 2016. “Ageing and Brain White Matter Structure in 3,513 UK Biobank Participants.” Nature Communications 7, no. 1: 13629. 10.1038/ncomms13629 PMC5172385 27976682

[hbm70142-bib-0043] de Kouchkovsky, I. , E. Fieremans , L. Fleysher , J. Herbert , R. I. Grossman , and M. Inglese . 2016. “Quantification of Normal‐Appearing White Matter Tract Integrity in Multiple Sclerosis: A Diffusion Kurtosis Imaging Study.” Journal of Neurology 263, no. 6: 1146–1155. 27094571 10.1007/s00415-016-8118-z PMC5369414

[hbm70142-bib-0020] Desikan, R. S. , F. Ségonne , B. Fischl , et al. 2006. “An Automated Labeling System for Subdividing the Human Cerebral Cortex on MRI Scans Into Gyral Based Regions of Interest.” NeuroImage 31, no. 3: 968–980. 16530430 10.1016/j.neuroimage.2006.01.021

[hbm70142-bib-0021] Does, M. D. , J. L. Olesen , K. D. Harkins , et al. 2019. “Evaluation of Principal Component Analysis Image Denoising on Multi‐Exponential MRI Relaxometry.” Magnetic Resonance in Medicine 81, no. 6: 3503–3514. 30720206 10.1002/mrm.27658 PMC6955240

[hbm70142-bib-0022] Dong, J. W. , I. O. Jelescu , B. Ades‐Aron , et al. 2020. “Diffusion MRI Biomarkers of White Matter Microstructure Vary Nonmonotonically With Increasing Cerebral Amyloid Deposition.” Neurobiology of Aging 89: 118–128. 32111392 10.1016/j.neurobiolaging.2020.01.009 PMC7314576

[hbm70142-bib-0023] Fadnavis, S. , J. Batson , and E. Garyfallidis . 2020. “Patch2Self: Denoising Diffusion MRI With Self‐Supervised Learning.” Advances in Neural Information Processing Systems 33: 16293–16303.

[hbm70142-bib-0024] Falangola, M. F. , J. H. Jensen , A. Tabesh , et al. 2013. “Non‐Gaussian Diffusion MRI Assessment of Brain Microstructure in Mild Cognitive Impairment and Alzheimer's Disease.” Magnetic Resonance Imaging 31, no. 6: 840–846. 23602730 10.1016/j.mri.2013.02.008 PMC5347444

[hbm70142-bib-0025] Gavish, M. , and D. L. Donoho . 2017. “Optimal Shrinkage of Singular Values.” IEEE Transactions on Information Theory 63, no. 4: 2137–2152.

[hbm70142-bib-0026] Griswold, M. A. , P. M. Jakob , R. M. Heidemann , et al. 2002. “Generalized Autocalibrating Partially Parallel Acquisitions (GRAPPA).” Magnetic Resonance in Medicine: An Official Journal of the International Society for Magnetic Resonance in Medicine 47, no. 6: 1202–1210. 10.1002/mrm.10171 12111967

[hbm70142-bib-0027] Grussu, F. , M. Battiston , J. Veraart , et al. 2020. “Multi‐Parametric Quantitative In Vivo Spinal Cord MRI With Unified Signal Readout and Image Denoising.” NeuroImage 116884: 116884. 10.1016/j.neuroimage.2020.116884 PMC7378937 32360689

[hbm70142-bib-0028] Gudbjartsson, H. , and S. Patz . 1995. “The Rician Distribution of Noisy Mri Data.” Magnetic Resonance in Medicine 34, no. 6: 910–914. 8598820 10.1002/mrm.1910340618 PMC2254141

[hbm70142-bib-0029] Hansen, B. , T. E. Lund , R. Sangill , and S. N. Jespersen . 2013. “Experimentally and Computationally Fast Method for Estimation of a Mean Kurtosis.” Magnetic Resonance in Medicine 69, no. 6: 1754–1760. 23589312 10.1002/mrm.24743

[hbm70142-bib-0030] Henriques, R. N. , A. Ianuş , L. Novello , J. Jovicich , S. N. Jespersen , and N. Shemesh . 2023. “Efficient PCA Denoising of Spatially Correlated Redundant MRI Data.” Imaging Neuroscience 1: 1–26. 10.1162/imag_a_00049 PMC12180759 40799709

[hbm70142-bib-0031] Hua, K. , J. Zhang , S. Wakana , et al. 2008. “Tract Probability Maps in Stereotaxic Spaces: Analyses of White Matter Anatomy and Tract‐Specific Quantification.” NeuroImage 39, no. 1: 336–347. 17931890 10.1016/j.neuroimage.2007.07.053 PMC2724595

[hbm70142-bib-0032] Jack, C. R., Jr. , M. A. Bernstein , N. C. Fox , et al. 2008. “The Alzheimer's Disease Neuroimaging Initiative (ADNI): MRI Methods. Journal of Magnetic Resonance Imaging: An Official Journal of the International Society for.” Magnetic Resonance in Medicine 27, no. 4: 685–691. 10.1002/jmri.21049 PMC2544629 18302232

[hbm70142-bib-0033] Jelescu, I. O. , and M. D. Budde . 2017. “Design and Validation of Diffusion MRI Models of White Matter.” Frontiers of Physics 5: 61. 10.3389/fphy.2017.00061 PMC5947881 29755979

[hbm70142-bib-0034] Jensen, J. H. , J. A. Helpern , A. Ramani , H. Lu , and K. Kaczynski . 2005. “Diffusional Kurtosis Imaging: The Quantification of Non‐Gaussian Water Diffusion by Means of Magnetic Resonance Imaging.” Magnetic Resonance in Medicine: An Official Journal of the International Society for Magnetic Resonance in Medicine 53, no. 6: 1432–1440. 10.1002/mrm.20508 15906300

[hbm70142-bib-0035] Jespersen, S. N. , J. L. Olesen , B. Hansen , and N. Shemesh . 2018. “Diffusion Time Dependence of Microstructural Parameters in Fixed Spinal Cord.” NeuroImage 182: 329–342. 28818694 10.1016/j.neuroimage.2017.08.039 PMC5812847

[hbm70142-bib-0086] Johnstone, I. M. 2007. “High Dimensional Statistical Inference and Random Matrices.” In Proceedings of the International Congress of Mathematicians Madrid, 307–333.

[hbm70142-bib-0037] Jones, D. K. , and M. R. I. Diffusion . 2010. Theory, Methods, and Application, xvi, 767. Oxford; New York: Oxford University Press.

[hbm70142-bib-0036] Jones, D. K. , and P. J. Basser . 2004. “‘Squashing Peanuts and Smashing Pumpkins’: How Noise Distorts Diffusion‐Weighted MR Data.” Magnetic Resonance in Medicine 52, no. 5: 979–993. 15508154 10.1002/mrm.20283

[hbm70142-bib-0038] Kamiya, K. , K. Kamagata , K. Ogaki , et al. 2020. “Brain White‐Matter Degeneration due to Aging and Parkinson Disease as Revealed by Double Diffusion Encoding.” Frontiers in Neuroscience—Switzerland 14: 584510. 10.3389/fnins.2020.584510 PMC7594529 33177985

[hbm70142-bib-0039] Kellner, E. , B. Dhital , V. G. Kiselev , and M. Reisert . 2016. “Gibbs‐Ringing Artifact Removal Based on Local Subvoxel‐Shifts.” Magnetic Resonance in Medicine 76, no. 5: 1574–1581. 26745823 10.1002/mrm.26054

[hbm70142-bib-0040] Kim, H.‐Y. 2016. “Statistical Notes for Clinical Researchers: Sample Size Calculation 1. Comparison of Two Independent Sample Means.” Restorative Dentistry & Endodontics 41, no. 1: 74–78. 26877994 10.5395/rde.2016.41.1.74 PMC4751211

[hbm70142-bib-0041] Kochunov, P. , L. E. Hong , E. L. Dennis , et al. 2022. “ENIGMA‐DTI: Translating Reproducible White Matter Deficits Into Personalized Vulnerability Metrics in Cross‐Diagnostic Psychiatric Research.” Human Brain Mapping 43, no. 1: 194–206. 32301246 10.1002/hbm.24998 PMC8675425

[hbm70142-bib-0042] Kornaropoulos, E. N. , S. Winzeck , T. Rumetshofer , et al. 2022. “Sensitivity of Diffusion MRI to White Matter Pathology: Influence of Diffusion Protocol, Magnetic Field Strength, and Processing Pipeline in Systemic Lupus Erythematosus.” Frontiers in Neurology 13: 837385. 35557624 10.3389/fneur.2022.837385 PMC9087851

[hbm70142-bib-0044] Kristo, G. , A. Leemans , B. de Gelder , M. Raemaekers , G.‐J. Rutten , and N. Ramsey . 2013. “Reliability of the Corticospinal Tract and Arcuate Fasciculus Reconstructed With DTI‐Based Tractography: Implications for Clinical Practice.” European Radiology 23: 28–36. 22868481 10.1007/s00330-012-2589-9

[hbm70142-bib-0045] Laguna, P. A. L. , A. J. Combes , J. Streffer , et al. 2020. “Reproducibility, Reliability and Variability of FA and MD in the Older Healthy Population: A Test‐Retest Multiparametric Analysis.” NeuroImage: Clinical 26: 102168. 32035272 10.1016/j.nicl.2020.102168 PMC7011084

[hbm70142-bib-0046] Larkman, D. J. , J. V. Hajnal , A. H. Herlihy , G. A. Coutts , I. R. Young , and G. Ehnholm . 2001. “Use of Multicoil Arrays for Separation of Signal From Multiple Slices Simultaneously Excited.” Journal of Magnetic Resonance Imaging: An Official Journal of the International Society for Magnetic Resonance in Medicine 13, no. 2: 313–317. 10.1002/1522-2586(200102)13:2<313::aid-jmri1045>3.0.co;2-w 11169840

[hbm70142-bib-0047] Lawrence, I. , and K. Lin . 1989. “A Concordance Correlation Coefficient To Evaluate Reproducibility.” Biometrics 45: 255–268. 2720055

[hbm70142-bib-0048] Lee, H. H. , D. S. Novikov , and E. Fieremans . 2021. “Removal of Partial Fourier‐Induced Gibbs (RPG) Ringing Artifacts in MRI.” Magnetic Resonance in Medicine 86: 2733–2750. 34227142 10.1002/mrm.28830 PMC9212190

[hbm70142-bib-0049] Lemberskiy, G. , S. Baete , J. Veraart , T. M. Shepherd , E. Fieremans , and D. S. Novikov . 2019. “Achieving Sub‐mm Clinical Diffusion MRI Resolution By Removing Noise During Reconstruction Using Random Matrix Theory.”

[hbm70142-bib-0050] Liu, X. , Y. Yang , J. Sun , et al. 2014. “Reproducibility of Diffusion Tensor Imaging in Normal Subjects: An Evaluation of Different Gradient Sampling Schemes and Registration Algorithm.” Neuroradiology 56: 497–510. 24609528 10.1007/s00234-014-1342-2

[hbm70142-bib-0051] Lu, H. , J. H. Jensen , A. Ramani , and J. A. Helpern . 2006. “Three‐Dimensional Characterization of Non‐Gaussian Water Diffusion in Humans Using Diffusion Kurtosis Imaging.” NMR in Biomedicine: An International Journal Devoted to the Development and Application of Magnetic Resonance in Vivo 19, no. 2: 236–247. 10.1002/nbm.1020 16521095

[hbm70142-bib-0052] Maggioni, M. , V. Katkovnik , K. Egiazarian , and A. Foi . 2012. “Nonlocal Transform‐Domain Filter for Volumetric Data Denoising and Reconstruction.” IEEE Transactions on Image Processing 22, no. 1: 119–133. 22868570 10.1109/TIP.2012.2210725

[hbm70142-bib-0053] Manjón, J. V. , P. Coupé , and A. Buades . 2015. “MRI Noise Estimation and Denoising Using Non‐Local PCA.” Medical Image Analysis 22, no. 1: 35–47. 25725303 10.1016/j.media.2015.01.004

[hbm70142-bib-0054] Manjón, J. V. , P. Coupé , L. Concha , A. Buades , D. L. Collins , and M. Robles . 2013. “Diffusion Weighted Image Denoising Using Overcomplete Local PCA.” PLoS One 8, no. 9: e73021. 24019889 10.1371/journal.pone.0073021 PMC3760829

[hbm70142-bib-0055] Manjon, J. V. , P. Coupe , L. Marti‐Bonmati , D. L. Collins , and M. Robles . 2010. “Adaptive Non‐Local Means Denoising of MR Images With Spatially Varying Noise Levels.” Journal of Magnetic Resonance Imaging 31, no. 1: 192–203. 20027588 10.1002/jmri.22003

[hbm70142-bib-0056] Manzano Patron, J. P. , S. Moeller , J. L. Andersson , K. Ugurbil , E. Yacoub , and S. N. Sotiropoulos . 2024. “Denoising Diffusion MRI: Considerations and Implications for Analysis.” Imaging Neuroscience 2: 1–29. 10.1162/imag_a_00060 PMC12224456 40800437

[hbm70142-bib-0057] McGraw, K. O. , and S. P. Wong . 1996. “Forming Inferences About Some Intraclass Correlation Coefficients.” Psychological Methods 1, no. 1: 30–46.

[hbm70142-bib-0058] Mirzaalian, H. , L. Ning , P. Savadjiev , et al. 2016. “Inter‐Site and Inter‐Scanner Diffusion MRI Data Harmonization.” NeuroImage 135: 311–323. 27138209 10.1016/j.neuroimage.2016.04.041 PMC5367052

[hbm70142-bib-0059] Moeller, S. , P. K. Pisharady , S. Ramanna , et al. 2021. “NOise Reduction With DIstribution Corrected (NORDIC) PCA in dMRI With Complex‐Valued Parameter‐Free Locally Low‐Rank Processing.” NeuroImage 226: 117539. 33186723 10.1016/j.neuroimage.2020.117539 PMC7881933

[hbm70142-bib-0060] Ning, L. , E. Bonet‐Carne , F. Grussu , et al. 2020. “Cross‐Scanner and Cross‐Protocol Multi‐Shell Diffusion MRI Data Harmonization: Algorithms and Results.” NeuroImage 221: 117128. 32673745 10.1016/j.neuroimage.2020.117128 PMC10243465

[hbm70142-bib-0061] Novikov, D. S. , E. Fieremans , S. N. Jespersen , and V. G. Kiselev . 2019. “Quantifying Brain Microstructure With Diffusion MRI: Theory and Parameter Estimation.” NMR in Biomedicine 32: e3998. 30321478 10.1002/nbm.3998 PMC6481929

[hbm70142-bib-0063] Novikov, D. S. , J. Veraart , I. O. Jelescu , and E. Fieremans . 2018. “Rotationally‐Invariant Mapping of Scalar and Orientational Metrics of Neuronal Microstructure With Diffusion MRI.” NeuroImage 174: 518–538. 29544816 10.1016/j.neuroimage.2018.03.006 PMC5949281

[hbm70142-bib-0062] Novikov, D. S. , V. G. Kiselev , and S. N. Jespersen . 2018. “On modeling.” Magnetic Resonance in Medicine 79, no. 6: 3172–3193. 29493816 10.1002/mrm.27101 PMC5905348

[hbm70142-bib-0064] Olesen, J. L. , A. Ianus , L. Østergaard , N. Shemesh , and S. N. Jespersen . 2023. “Tensor Denoising of Multidimensional MRI Data.” Magnetic Resonance in Medicine 89, no. 3: 1160–1172. 36219475 10.1002/mrm.29478 PMC10092037

[hbm70142-bib-0065] Pogosbekian, E. , I. N. Pronin , N. Zakharova , et al. 2021. “Feasibility of Generalised Diffusion Kurtosis Imaging Approach for Brain Glioma Grading.” Neuroradiology 63: 1241–1251. 33410948 10.1007/s00234-020-02613-7 PMC8295088

[hbm70142-bib-0085] Pruessmann, K. P. , M. Weiger , M. B. Scheidegger , and P. Boesiger . 1999. “SENSE: Sensitivity Encoding for Fast MRI.” Magnetic Resonance in Medicine 42, no. 5: 952–962. 10542355

[hbm70142-bib-0066] Setsompop, K. , B. A. Gagoski , J. R. Polimeni , T. Witzel , V. J. Wedeen , and L. L. Wald . 2012. “Blipped‐Controlled Aliasing in Parallel Imaging for Simultaneous Multislice Echo Planar Imaging With Reduced g‐Factor Penalty.” Magnetic Resonance in Medicine 67, no. 5: 1210–1224. 21858868 10.1002/mrm.23097 PMC3323676

[hbm70142-bib-0067] Smith, S. M. , M. Jenkinson , M. W. Woolrich , et al. 2004. “Advances in Functional and Structural MR Image Analysis and Implementation as FSL.” NeuroImage 23: S208–S219. 15501092 10.1016/j.neuroimage.2004.07.051

[hbm70142-bib-0068] Sodickson, D. K. , and W. J. Manning . 1997. “Simultaneous Acquisition of Spatial Harmonics (SMASH): Fast Imaging With Radiofrequency Coil Arrays.” Magnetic Resonance in Medicine 38, no. 4: 591–603. 9324327 10.1002/mrm.1910380414

[hbm70142-bib-0069] Sprenger, T. , J. I. Sperl , B. Fernandez , et al. 2016. “Bias and Precision Analysis of Diffusional Kurtosis Imaging for Different Acquisition Schemes.” Magnetic Resonance in Medicine 76, no. 6: 1684–1696. 26822349 10.1002/mrm.26008

[hbm70142-bib-0071] St‐Jean, S. , P. Coupé , and M. Descoteaux . 2016. “Non Local Spatial and Angular Matching: Enabling Higher Spatial Resolution Diffusion MRI Datasets Through Adaptive Denoising.” Medical Image Analysis 32: 115–130. 27082655 10.1016/j.media.2016.02.010

[hbm70142-bib-0070] Stauffer, E.‐M. , R. A. Bethlehem , V. Warrier , et al. 2021. “Grey and White Matter Microstructure Is Associated With Polygenic Risk for Schizophrenia.” Molecular Psychiatry 26, no. 12: 7709–7718. 34462574 10.1038/s41380-021-01260-5 PMC8872982

[hbm70142-bib-0072] Sudlow, C. , J. Gallacher , N. Allen , et al. 2015. “UK Biobank: An Open Access Resource for Identifying the Causes of a Wide Range of Complex Diseases of Middle and Old Age.” PLoS Medicine 12, no. 3: e1001779. 25826379 10.1371/journal.pmed.1001779 PMC4380465

[hbm70142-bib-0073] Szczepankiewicz, F. , J. Lätt , R. Wirestam , et al. 2013. “Variability in Diffusion Kurtosis Imaging: Impact on Study Design, Statistical Power and Interpretation.” NeuroImage 76: 145–154. 23507377 10.1016/j.neuroimage.2013.02.078

[hbm70142-bib-0074] Tian, C. , Y. Xu , Z. Li , W. Zuo , L. Fei , and H. Liu . 2020. “Attention‐Guided CNN for Image Denoising.” Neural Networks 124: 117–129. 31991307 10.1016/j.neunet.2019.12.024

[hbm70142-bib-0075] Tian, Q. , Z. Li , Q. Fan , et al. 2022. “SDnDTI: Self‐Supervised Deep Learning‐Based Denoising for Diffusion Tensor MRI.” NeuroImage 253: 119033. 35240299 10.1016/j.neuroimage.2022.119033 PMC9511973

[hbm70142-bib-0076] Tournier, J.‐D. , R. Smith , D. Raffelt , et al. 2019. “MRtrix3: A Fast, Flexible and Open Software Framework for Medical Image Processing and Visualisation.” NeuroImage 202: 116137. 31473352 10.1016/j.neuroimage.2019.116137

[hbm70142-bib-0077] Van Essen, D. C. , S. M. Smith , D. M. Barch , et al. 2013. “The WU‐Minn Human Connectome Project: An Overview.” NeuroImage 80: 62–79. 23684880 10.1016/j.neuroimage.2013.05.041 PMC3724347

[hbm70142-bib-0079] Veraart, J. , D. S. Novikov , D. Christiaens , B. Ades‐Aron , J. Sijbers , and E. Fieremans . 2016. “Denoising of Diffusion MRI Using Random Matrix Theory.” NeuroImage 142: 384–396. 10.1016/j.neuroimage.2016.08.016 PMC5159209 27523449

[hbm70142-bib-0078] Veraart, J. , E. Fieremans , and D. S. Novikov . 2016. “Diffusion MRI Noise Mapping Using Random Matrix Theory.” Magnetic Resonance in Medicine 76, no. 5: 1582–1593. 26599599 10.1002/mrm.26059 PMC4879661

[hbm70142-bib-0080] Veraart, J. , J. Sijbers , S. Sunaert , A. Leemans , and B. Jeurissen . 2013. “Weighted Linear Least Squares Estimation of Diffusion MRI Parameters: Strengths, Limitations, and Pitfalls.” NeuroImage 81: 335–346. 23684865 10.1016/j.neuroimage.2013.05.028

[hbm70142-bib-0081] Vollmar, C. , J. O'Muircheartaigh , G. J. Barker , et al. 2010. “Identical, but Not the Same: Intra‐Site and Inter‐Site Reproducibility of Fractional Anisotropy Measures on Two 3.0 T Scanners.” NeuroImage 51, no. 4: 1384–1394. 20338248 10.1016/j.neuroimage.2010.03.046 PMC3163823

[hbm70142-bib-0082] Walsh, D. O. , A. F. Gmitro , and M. W. Marcellin . 2000. “Adaptive Reconstruction of Phased Array MR Imagery.” Magnetic Resonance in Medicine: An Official Journal of the International Society for Magnetic Resonance in Medicine 43, no. 5: 682–690. 10.1002/(sici)1522-2594(200005)43:5<682::aid-mrm10>3.0.co;2-g 10800033

[hbm70142-bib-0083] Zhang, F. , L. Ning , L. J. O'Donnell , and O. Pasternak . 2019. “MK‐Curve‐Characterizing the Relation Between Mean Kurtosis and Alterations in the Diffusion MRI Signal.” NeuroImage 196: 68–80. 30978492 10.1016/j.neuroimage.2019.04.015 PMC6592693

[hbm70142-bib-0084] Zhao, Y. , Z. Yi , L. Xiao , et al. 2022. “Joint Denoising of Diffusion‐Weighted Images via Structured Low‐Rank Patch Matrix Approximation.” Magnetic Resonance in Medicine 88, no. 6: 2461–2474. 36178232 10.1002/mrm.29407

