# Supplementary material for: Denoising Improves Cross‐Scanner and Cross‐Protocol Test–Retest Reproducibility of Diffusion Tensor and Kurtosis Imaging
Source: Hum Brain Mapp. 2025 Mar 7;46(4):e70142. doi: 10.1002/hbm.70142 (PMC11885890; doi:10.1002/hbm.70142)
Supplement: Supplementary file 1 — Data S1. Supporting Information. [file HBM-46-e70142-s001.docx]

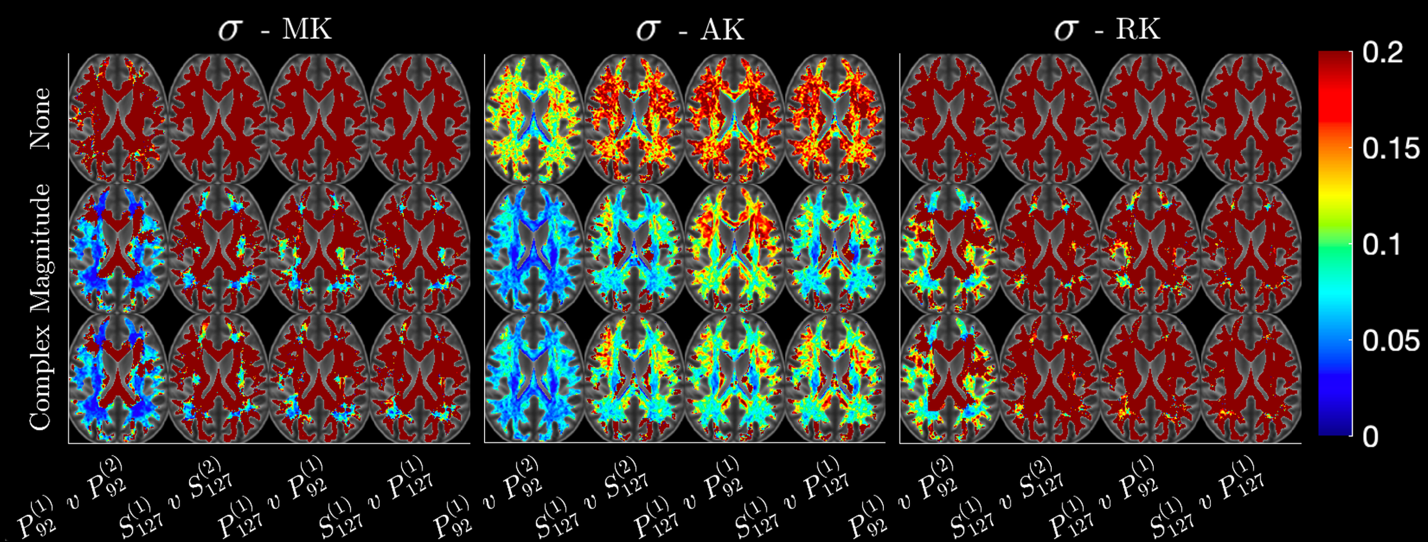


Figure S1: Maps of $\sigma_{x}$ in WM without normalization averaged over all 20 subjects for K. Error maps were computed in each subject’s space and then warped into a common space prior to averaging. For each parameter left two columns show within-scan repeatability ($P_{92}^{\left( 1 \right)}$vs $P_{92}^{\left( 2 \right)}$and $S_{127}^{\left( 1 \right)}$^)^ vs $S_{127}^{\left( 2 \right)}$ ), third column shows cross-protocol variability ($P_{92}^{\left( 1 \right)}$vs $P_{127}^{\left( 1 \right)}$), and fourth column shows cross-scanner variability ($P_{127}^{\left( 1 \right)}$ vs $S_{127}^{\left( 1 \right)}$).


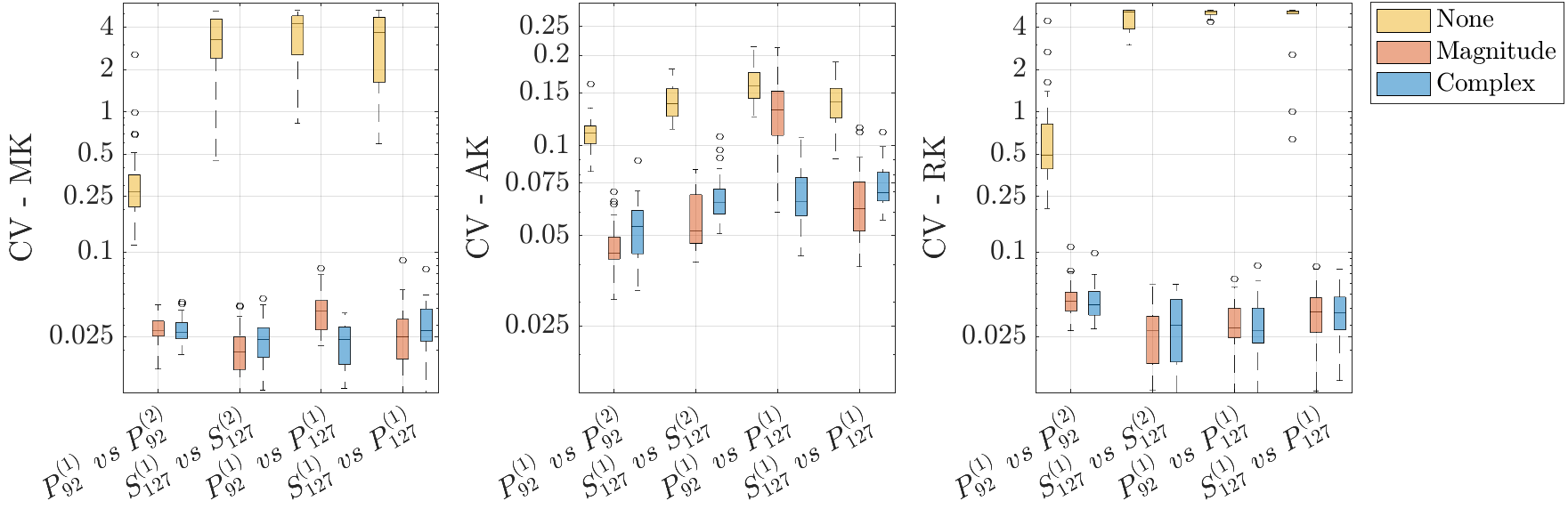


Figure S2: Box plots for 20 subjects where CV for *K* maps were pooled over ROIs. Plots show $\sigma_{x}/\mu_{x}$ after pooling DTI and DKI parameters over whiter matter (PLIC, ALIC) over all subjects. X-axes show each of the four comparisons: within-scan variability ($P_{92}^{\left( 1 \right)}$ vs $P_{92}^{\left( 2 \right)}$and $S_{127}^{\left( 1 \right)}$vs $S_{127}^{\left( 2 \right)}$ ), Cross-protocol variability ($P_{92}^{\left( 1 \right)}$vs $P_{127}^{\left( 1 \right)}$), and cross-scanner variability ($P_{127}^{\left( 1 \right)}$vs $S_{127}^{\left( 1 \right)}$).


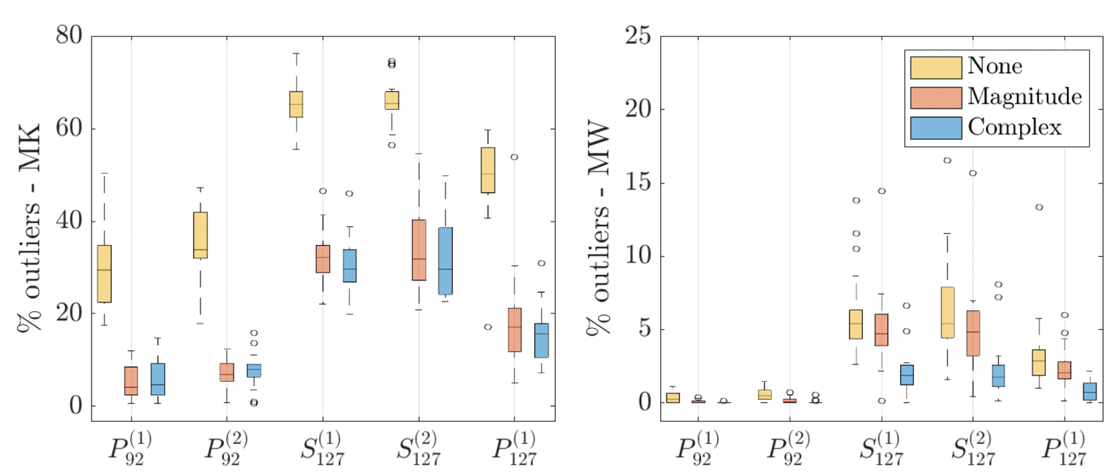


Figure S3: Percentage of outliers in MK and MW with each denoising method in the PLIC. The percentage of K outliers reaches 70% on the Skyra system, while the percentage of W outliers is consistently less than 10% on both scanners and the lowest after denoising.


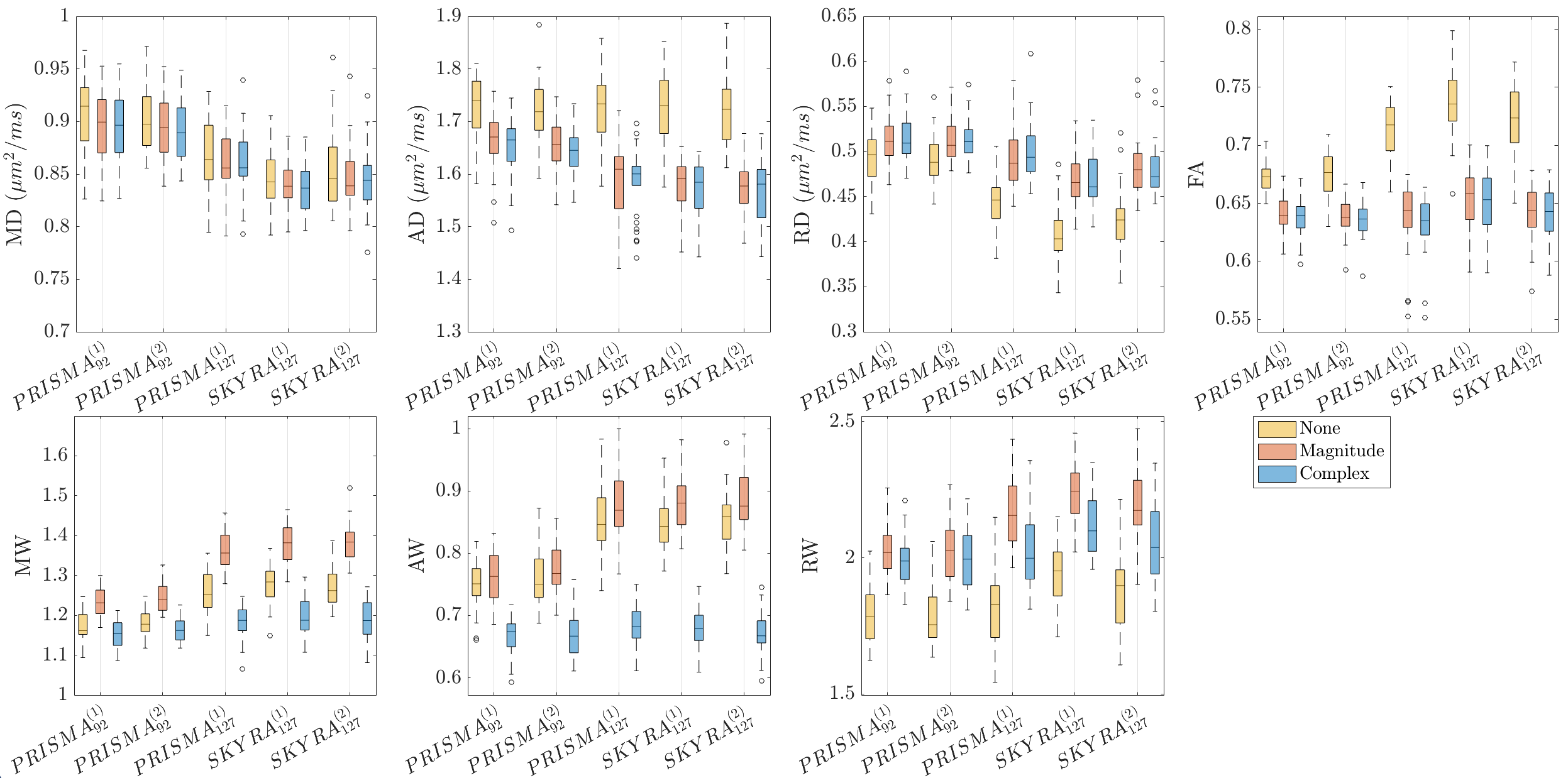
Figure S4: Boxplots of average ROI-values in white matter for each DTI parameters over all 20 subjects. Parameters here are derived from a DKI fit rather than a DTI fit.

Table S1: 20 Subject mean ROI-wise coefficients of variation for each DTI and DKI parameter and denoising type evaluated in four regions: Splenium of the corpus callosum, posterior limb of the internal capsule (PLIC), Anterior Corona Radiata (ACR), and thalamus. We show coefficients of variation for each of the three comparisons: within-scan variability ($P_{92}^{\left( 1 \right)}$ vs $P_{92}^{\left( 2 \right)}$and $S_{127}^{\left( 1 \right)}$vs $S_{127}^{\left( 2 \right)}$ ), Cross-protocol variability ($P_{92}^{\left( 1 \right)}$vs $P_{127}^{\left( 1 \right)}$), and cross-scanner variability ($P_{127}^{\left( 1 \right)}$vs $S_{127}^{\left( 1 \right)}$).
